# Identification of unique rectal cancer-specific subtypes

**DOI:** 10.1038/s41416-024-02656-0

**Published:** 2024-03-26

**Authors:** Batuhan Kisakol, Anna Matveeva, Manuela Salvucci, Alexander Kel, Elizabeth McDonough, Fiona Ginty, Daniel B. Longley, Jochen H. M. Prehn

**Affiliations:** 1 Department of Physiology and Medical Physics, Royal College of Surgeons in Ireland, Dublin, 2 Ireland; 2 Centre for Systems Medicine, Royal College of Surgeons in Ireland, Dublin, 2 Ireland; 3 geneXplain GmbH, Wolfenbüttel, Germany; 4 GE Research, Niskayuna, NY 12309 USA; 5 Centre for Cancer Research & Cell Biology, Queen’s University Belfast, Belfast, Northern Ireland UK

**Keywords:** Tumour biomarkers, Prognostic markers, Rectal cancer, Single-cell imaging, Cancer genomics

## Abstract

**Background:**

Existing colorectal cancer subtyping methods were generated without much consideration of potential differences in expression profiles between colon and rectal tissues. Moreover, locally advanced rectal cancers at resection often have received neoadjuvant chemoradiotherapy which likely has a significant impact on gene expression.

**Methods:**

We collected mRNA expression profiles for rectal and colon cancer samples (*n* = 2121). We observed that (i) Consensus Molecular Subtyping (CMS) had a different prognosis in treatment-naïve rectal vs. colon cancers, and (ii) that neoadjuvant chemoradiotherapy exposure produced a strong shift in CMS subtypes in rectal cancers. We therefore clustered 182 untreated rectal cancers to find rectal cancer-specific subtypes (RSSs).

**Results:**

We identified three robust subtypes. We observed that RSS1 had better, and RSS2 had worse disease-free survival. RSS1 showed high expression of MYC target genes and low activity of angiogenesis genes. RSS2 exhibited low regulatory T cell abundance, strong EMT and angiogenesis signalling, and high activation of TGF-β, NF-κB, and TNF-α signalling. RSS3 was characterised by the deactivation of EGFR, MAPK and WNT pathways.

**Conclusions:**

We conclude that RSS subtyping allows for more accurate prognosis predictions in rectal cancers than CMS subtyping and provides new insight into targetable disease pathways within these subtypes.

## Background

Colorectal cancer is the third most common cancer worldwide. When considered separately colon cancer is the fourth and rectal cancer is the eighth most common cancer [1]. Rectal and colon cancers exhibit histological and anatomical differences which have been associated with different clinical outcomes [2, 3]. While stage III and high-risk stage II colon cancer patients are treated with adjuvant chemotherapy post-resection [4], locally advanced rectal cancers are routinely treated both in neoadjuvant and adjuvant settings. Neoadjuvant chemoradiotherapy (CRT) is performed in stage 3 and high-risk stage 2 rectal cancers to shrink the tumour and improve the effectiveness of surgical resection [5].

Unsupervised, transcriptome-based molecular subtyping of colorectal cancers has revealed new insights into the heterogeneity of colorectal cancer and the underlying disease biology within subtypes [6]. ‘Consensus molecular subtypes’ (CMS) defined by Guinney et al. [7] were established subsequent to several previous subtyping studies [8–13] and employed Markov clustering on the similarity network of the previously identified clusters to define four CMS. However, colon cancer samples comprised the vast majority (85%) of the study samples [14]. Moreover, rectal cancers at resection may have already been subjected to neoadjuvant CRT, which is likely to induce significant alterations in gene expression profiles [5].

Systematic and specific subtyping of treatment-naïve rectal cancers has so far not been performed. Therefore, by focusing on untreated rectal cancer samples, we refined previous subtyping efforts and identified three robust RSS, RSS1/2/3. These subtypes displayed promising results regarding their prognostic power in rectal cancer. We demonstrated differences in disease biology between RSS subtypes using an in-house cohort of rectal cancers analysed by a multiplex imaging platform. Our findings shed light on the molecular features of rectal cancers and suggest novel subtyping could benefit cancer prognosis.

## Methods

### Samples

In total, we gathered 2121 microarray samples from 18 different studies in the GEO platform and 608 RNA-sequencing samples from TCGA. However, microarray samples contained duplicate entries which were submitted in separate studies under different IDs. These duplicate samples were detected using the md5 checksum values of the raw files of each sample and single entries were kept after removing the duplicates (*n* = 1820, Supplementary Table 1A–D).

### Training and validation datasets

**Fig. 1:**
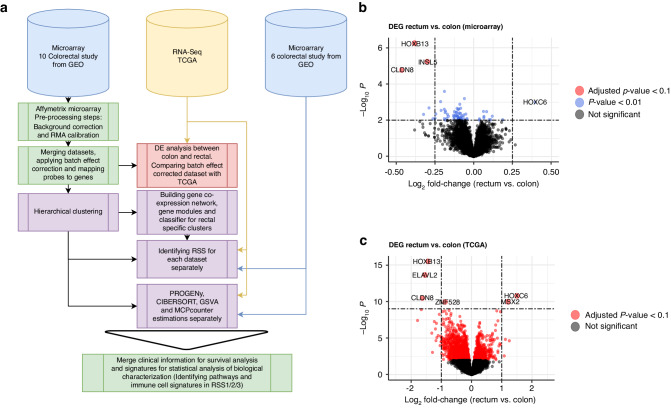
The overall workflow of the analyses and colon vs rectum comparison. **a** Workflow of the data processing steps. Raw files (.CEL) were downloaded from 16 different colorectal studies in the Gene Expression Omnibus (GEO) database. 10 datasets were processed following the same steps. After pre-processing, they were merged and batch effect correction was applied. Hierarchical clustering in rectal samples (*n* = 182) in these datasets was calculated to define rectal-specific subtypes (RSS). Then, by generating gene co-expression networks and identifying gene modules, we developed a classifier to predict RSSs in different datasets. 6 microarray datasets and TCGA RNA-seq samples were separated initially due to the different technology platforms there were originated from. RSSs were calculated in these datasets separately after the classifier was developed. Volcano plots of differential expression analysis show that top differentially expressed genes between the colon and rectum in microarray (**b**) and RNA-Seq (**c**) datasets are similar. HOXB13, HOXC6 and CLDN8 were among the most DEGs in both cohorts. Different thresholds (*p-*value and fold-change) were used to demonstrate the differential expression in microarray and RNA-Seq due to the unbalanced distribution of colon/rectal ratio and lower sensitivity in microarray datasets.

We designed a large cohort for our clustering analysis by merging the microarray datasets. However, since these datasets were generated in different laboratories using different technologies, we needed to address and solve the batch effect problem in the merging process. Each probe set has unique identifiers on different technologies. Moreover, the number of gene measurements varies depending on the microarray platform. Therefore, pre-processing and merging in these steps were done on only 10 GEO datasets which all used the same technology (Affymetrix) and similar platform versions (Human Genome U133A Array or Human Genome U133 Plus 2.0 Array). Raw CEL files were downloaded from GEO and processed with standard RMA normalisation for these 10 datasets. After removing the duplicate values in the training cohort we pooled 1291 samples (Colon = 1109 and Rectal = 182, Supplementary Table 1A). Clinical information regarding the training datasets can be found in Supplementary Table 2A. In the rest of the analysis, rectal samples (*n* = 182) of this cohort were used as training for rectal-specific subtyping. The rest of the microarray samples (Supplementary Table 1B, C) and TCGA-COAD-READ RNA-Seq samples (Supplementary Table 1D) were used as a validation/exploration cohort (Fig. 1). TCGA-COAD-READ RNA-Seq samples also were used as a validation for colon vs rectal comparison. Finally, Supplementary Table 3 provides information on the datasets utilised for specific types of analyses along with the total number of samples analysed.

### Gene Co-expression networks

Since the number of genes targeted in each microarray study was different, building a model using the gene expressions as features was not feasible. Therefore, we developed a gene co-expression network and further categorised genes into modules.

To narrow down which genes were affecting these clusters, we ran an ANOVA on RSS1 vs RSS2, RSS2 vs RSS3 and RSS1 vs RSS3, and then gathered genes that had statistical significance (FDR < 0.05) on any of the pairwise comparisons (5989 genes in total). A gene co-expression network was created on the filtered 5989 genes following the approach detailed in Song et al. [15]. For this purpose, the Spearman correlation for each pair in selected genes was calculated and modified from our similarity matrix into an adjacency matrix. Based on the adjacency matrix, we calculated connectivity scores for each gene. Median connectivity was found as 0.1 and we restricted the analysis by only using genes showing high connectivity (>0.1).

Then, we calculated topological overlap matrix dissimilarity based on our restricted adjacency matrix. After finalising the gene expression network, we calculated hierarchical clustering on genes with dynamic tree cutting. Similar to the method used by Budinska et al. [9], we ran dynamic tree clustering from *k* = 5 to *k* = 101 and assigned genes to their largest cluster.

Lastly, we identified 88 gene modules comprising a total of 3200 genes. To identify pathways with each gene module, we applied the topGO [16] tool on each gene module. We eliminated genes that were not in any significant pathways and removed modules if they fell below the minimum 5 genes after filtering. At this last step, we gathered 54 gene modules with 1599 genes.

### Building a classifier

We built a classifier to explore RSS in other datasets. The classifier used log2 normalised gene expression counts and aggregated them into 54 pre-defined gene modules. Using the median values of 54 gene modules it classified the RSSs. XgBoost classifier algorithm was used with 5-fold cross-validation in the training dataset using median values for genes in modules. 90 + % accuracies were achieved in each cross-validation fold. We found the optimal number of clusters as three in our cohort using the ‘deltaK’ method described by Monti et al. [17]. Silhouette plots and principal component analysis were done to demonstrate the robustness of the clustering. We successfully reproduced RSS in validation and test cohorts regardless of the gene coverage of the datasets.

### Survival analysis

**Fig. 2:**
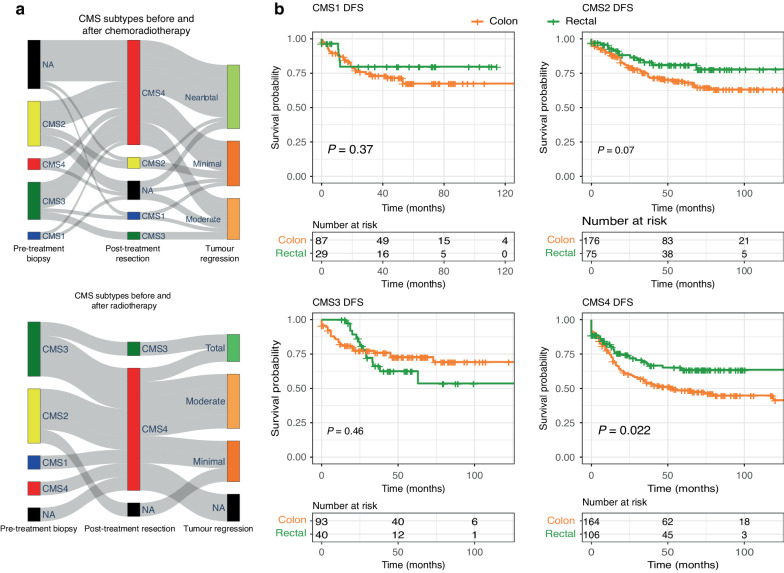
Effect of chemoradiotherapy on subtyping and survival patterns. **a** Sankey plots of the CMS shift after radiotherapy (GSE56699 study, upper plot) and after chemotherapy (GSE94104, lower Sankey plot) point to a significant shift in subtypes after CRT. Neoadjuvant therapy in rectal cancers causes a mesenchymal transition in molecular subtypes. Tumour regression grades in the GSE94104 study represent: ‘Minimal’: “TRG1 - Minor Regression”; ‘Moderate’: “Significant Regression”; NearTotal: “Small Tumour Left”. TRG classes in the GSE56699 study were: ‘Total’: “TRG 1 (Mandard): Complete Response, No tumour”; ‘Minimal: “TRG 4 (Mandard): Residual Tumour > Fibrosis”; ‘Moderate’: “TRG 3 (Mandard): Fibrosis > Residual Tumour. **b** Kaplan–Meier plots for colon vs pre-treatment naïve rectal cancer samples in different CMS subgroups show a significant difference between colon and rectal cancers in the CMS4 subtype. Overall rectal cancers have better disease-free survival than colons except for CMS3.

**Fig. 3:**
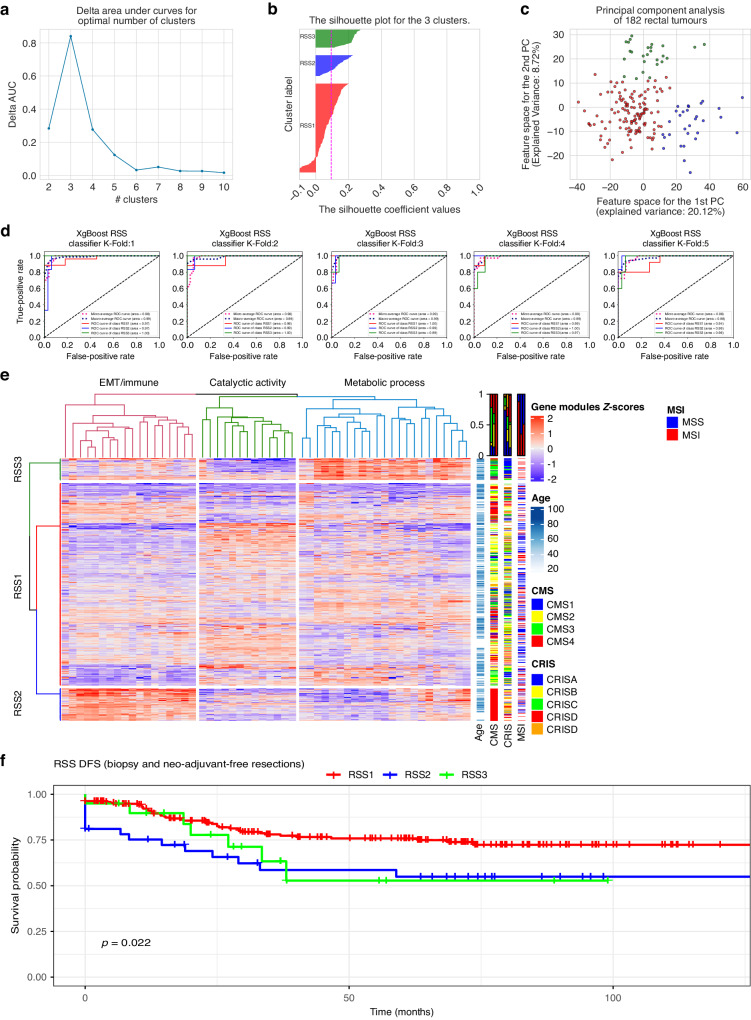
Identification and molecular characteristics of RSS. **a** Delta plots to select the optimal number of clusters. 3 clusters were found to be the optimum number of clusters. **b** Silhouette plots of the hierarchical clusters on the exploration dataset (*n* = 182). **c** PCA plot of the hierarchical clusters on the exploration dataset. **d** 5-Fold validation and ROC Curve plots of the RSS classifier model on the exploration dataset. Accuracies were calculated based on the one-vs-all approach for each group. In each fold, 90 + % accuracies were achieved. **e** Heatmap of the gene modules and distribution of RSS, CMS, CRIS, MSI and age. Rows represent the patients, RSS groups on the left and their clinical information on the right. Columns represent the median value of each gene module. The top of the column shows the biological/molecular characteristics of the modules. Heatmap representation based on all the rectal samples (*n* = 870, Supplementary Table 3) after the RSS classification is applied to the rest of the microarray and RNA-Seq samples. **f** Kaplan–Meier survival plots of the rectal specific subtypes when classification derived from pre-treatment samples. RSS1 represents the best disease-free survival group while RSS2 has the worst disease-free survival. The *p*-value (0.022) is based on the log-rank test.

250 rectal and 520 colon samples were used for disease-free survival analysis from multiple microarray datasets (Fig. 2 and Supplementary Table 3) for CMS comparison. 271 Rectal samples were available with DFS information and RSS classification (Fig. 3). Kaplan-Meier (KM) plots and Cox regression models are generated using the ‘survminer’ package in R. If the “Stage” (and “T stage”) had missing values in the clinical files the patient was classified as Stage 3 as long as “‘N stage’ >0” and “‘M stage’=0”.

### CMS and CRIS classifications

We classified CMS molecular subtypes with CMSCaller [18] and CRIS with CRISClassifier [19].

### Master regulators

To identify master regulators in defined subtypes, we treated each subtype as a separate disease and compared them to normal samples. Only the GSE87211 dataset was used for this analysis, as it had the most normal tissues and rectal samples. This cohort was not a part of the exploration cohort where clustering was performed. We used GeneXplain’s TRANSPATH [20, 21] database to search master regulators by identifying global signal transduction networks. After finding the differentially expressed genes on clusters against normal tissues, we filtered genes with FDR < 10^−6^ and |FoldChange | > 2. Supplementary Table 5 shows the number of master regulators in each cluster.

### Immune cell analysis

CIBERSORT [22] and MCPcounter [23] analytical tools were used to infer immune cell fractions. Estimations were done on the training dataset (batch effect corrected 182 treatment-naïve rectal samples), TCGA and 6 validation microarray datasets from GEO separately. Leukocyte gene signature matrix (LM22) was used for CIBERSORT analysis. This gene signature matrix includes 547 genes which CIBERSORT uses for deconvolution and identifies 22 different immune cell populations including 7 different types of T cells, naïve and memory B cell, plasma cells and myeloid. Similar to CIBERSORT, the MCPcounter algorithm estimates the population abundance of immune and stromal cells. Additionally, we gathered fibroblasts and endothelial cell populations using the MCPcounter algorithm. Tumour purity, stromal and immune scores were also generated using the ESTIMATE [24] algorithm.

### PROGENy and GSVA

Pathway activity scores were calculated with the PROGENy [25] tool. PROGENy model weights each gene to 14 curated pathways. Depending on the *z*-score of gene expressions, it calculates the pathway response scores. We separately estimated the activity scores of 14 pathways using PROGENy on the microarray and RNA-Seq datasets. Gene Set Variation Analysis (GSVA) was also done on the rectal samples using the GSVA package in R [26]. Colon cancer stem cells [27] and other selected gene sets can be found in Supplementary Table 5.

### CELL DIVE

Multiplexed immunofluorescence (MxIF) staining was performed on 18 rectal tumour tissue microarrays using Cell DIVE technology. The protocols regarding the staining and the imaging are described in detail by Gerdes et al. [28, 29]. The FFPE slides were deparaffinized, underwent a two-step antigen retrieval procedure, were stained with DAPI, and images were taken in each of the interest channels to gather background tissue autofluorescence. Segmentation in the epithelial and stromal content was done using antibody stains against DAPI, pan-cytokeratin (PCK-26), S6 ribosomal protein, and Na^+^/K^+^-ATPase (Sodium-potassium). Then, single cell-level expressions of proteins with spatial coordinates on the images were generated. CD3, CD4, CD8, CD20, FOXP1 and PD1 expressions were used to identify immune cells in the images. We analysed the expression levels of 56 markers (Supplementary Table 4) with 162,296 cells from 52 TMA cores and 18 pre-treatment naïve rectal cancer patients. Image processing, quality control, and antibodies used on these samples are described by Lindner et al. [30] in depth. Virtual HE images were generated as described by Gerdes et al. [28, 29].

## Results

### Colon and rectal cancers differ in their gene expression profiles

The first aim of our study was to identify differences in gene expression levels between colon and rectal cancer samples. Differential expression (DE) analysis results on the merged (training, *n* = 1291) microarray datasets were also compared to TCGA RNA-sequencing (RNA-Seq) data of colon and rectal samples (validation, *n* = 608). Although the number of significant DE genes was higher in RNA-Seq samples than in batch-effect corrected microarray datasets, the top differentially expressed genes (DEG) were similar in both analyses (Fig. 1). The top two genes identified to be differentially expressed between rectal and colon cancers were HOX family genes: HOXB13 was upregulated and HOXC6 downregulated in the rectal cancer samples.

### Differences in the prognosis of CMS subtyping performed on colon and pre-treatment-naïve rectal cancer tissue

From the outset of our study, we hypothesised that neoadjuvant CRT changes gene expression in rectal cancer tissues and that this may impact the CMS subtype assigned. We therefore next investigated how CMS subtypes changed after neoadjuvant therapy in rectal cancers. Four separate gene expression datasets were analysed (Supplementary Table 3). We compared CMS status in matching pre-treatment biopsy and post-treatment resection samples. Of note, we observed that more than half of the samples shifted to CMS4 after patients received chemoradiotherapy or radiotherapy in all four separate studies (Fig. 2a and Supplementary Fig. 1). Samples that changed to CMS4 also showed a significant increase in gene expression profiles of fibroblasts (Supplementary Fig. 2A).

We next investigated possible prognostic differences of CMS subtyping between colon and rectal cancers when the CMS classification was only derived from pre-treatment naïve rectal cancer samples (*n* = 770, Fig. 2b, Supplementary Table 1A−D, Supplementary Table 3). We observed a significant difference between colon and rectal CMS4 tumours in prognosis. Rectal cancers showed a better prognosis in CMS4 compared to colon cancers (*p*-value: 0.022, Fig. 2b). We discovered a similar trend in CMS2 where rectal tumours tended to have a better prognosis (*p*-value: 0.07, Fig. 2b). We observed the same better rectal tumour prognosis in overall survival analysis as well (Supplementary Fig. 2B).

Isella et al. [19] developed colorectal cancer intrinsic subtype (CRIS) signatures that were derived solely from cancer cell-related genes. We therefore also performed a separate analysis by CRIS subtyping of pre-treatment naïve rectal cancer samples. Kaplan−Meier analysis between the colon and rectal cancers indicated that CRIS-B and CRIS-E subtypes had better DFS in rectal cancers compared to colon cancers. CRIS-D tumours showed better overall survival in the rectal compared to colon cancers (Supplementary Fig. 3). In the two other CRIS subtypes (A and C), rectal cancers had a similar prognosis when compared to colon cancers.

### Identification of three rectal-specific molecular subtypes in rectal cancer with prognostic information

Above, we demonstrated that CMS and CRIS subtypes in rectal cancers had different prognostic patterns than in colon cancers, suggesting differences in disease biology that were not captured by the existing subtypes. We, therefore, extended our analysis to devise a rectal-specific subtyping method, again focusing only on pre-treatment naïve samples. Using a curated microarray of 182 rectal cancer samples (Supplementary Table 1A), we identified three major subtypes in the treatment-naïve rectal cancers. Figure 3a−d demonstrates the robustness of the clustering and accuracies of the classifier we built to identify RSS in the different datasets used.

Figure 3e presents how these RSS correspond to existing subtyping methods as well as other clinical features such as age, sex, and stage. Almost all of the RSS2 samples were classified as CMS4 (>99%), but interestingly not all CMS4 samples were classified as RSS2. RSS3 was mainly composed of CMS3 and CRIS-A, while RSS1 exhibited a more mixed composition compared to RSS2 and RSS3. We next examined survival profiles in these subtypes and found that RSS2 had the worst disease-free survival among the three groups and RSS1 had the best survival (Fig. 3f). Similar to a shift to CMS4 after neoadjuvant CRT, we also observed a change from RSS1 to RSS2 after neoadjuvant therapy (Supplementary Fig. 4A, B). Degree of differentiation was available for a subset of the rectal tumours analysed, and correlation with RSS subtypes is shown in Supplementary Table 2B.

### Molecular characterisation of the three rectal-specific molecular subtypes

**Fig. 4:**
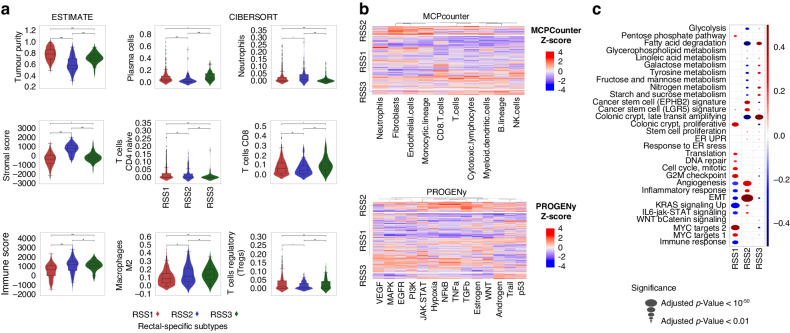
Immune landscape, pathway, and gene set analysis of RSS. **a** Boxplots of immune, stroma and tumour purity scores by ESTIMATE are in the first column. CIBERSORT results are shown in the second and third columns. Significant differences are observed in tumour purity, immune score, and stromal scores between subtypes in ESTIMATE results. The CIBERSORT algorithm demonstrates the immune cell infiltrations in different subtypes. Plasma cells, CD4 + T cells, CD8 + T cells, regulatory T cells (Tregs), M2 macrophages, and neutrophils are among the most distinguished immune cell signatures between RSSs. Star annotations regarding the *p*-values are as follows: “****”*p* < 0.0001; “***”*p* < 0.001; “**”*p* < 0.01; “*”*p* < 0.05; “ns”: *P* > 0.05. **b** MCPcounter immune signatures are shown in the first heatmap coloured with *z*-scores PROGENy pathway activity scores are in the second heatmap, coloured with *z*-scores (Blue- lower enrichment/activation and Red- higher enrichment/activation). High fibroblast and endothelial cell enrichments in RSS2 can be seen in the first heatmap. TGFβ, NFκB and TNFα activities in RSS2, and MAPK, WNT and EGFR deactivations in RSS3 are the most apparent results. **c** GSVA dot-plots of selected genesets, colours represent the fold-change, and the sizes of the dots represent the *p*-value (inversely). EMT and angiogenesis-related genes are highly expressed in RSS2 compared to the others. Whereas expressions of immune and inflammatory response genes are found to be lower in RSS1. All three graphs are based on all rectal samples, microarray and RNA-Seq together (*n* = 870, Supplementary Table 3).

Tumour purity and stromal/immune scores, calculated with ESTIMATE, of the RSSs revealed that RSS1 had high tumour purity and low immune score. RSS2/RSS3 were associated with high immune scores and RSS2 with a high stromal score (Fig. 4a). We further investigated the tumour infiltrating immune cells with CIBERSORT and MCPcounter. CIBERSORT results show that RSS1 had a lower mast cell and macrophage (M2) frequency than the other two groups. RSS2 exhibits low plasma cells and regulatory T cells compared to RSS1. RSS3 displayed a high prevalence of CD8 + T cells and B cell lineage, and lower abundance in CD4 + T cells, neutrophils and eosinophils (Fig. 4a, b and Supplementary Fig. 5A). MCPcounter results also suggested high fibroblasts and endothelial cells in RSS2. Moreover, high NK and B lineage levels were observed in RSS3 and low T cell, cytotoxic lymphocytes, and monocytic lineages were related to RSS1. Lastly, one of the characteristics of RSS3 was microsatellite instability.

Next, we determined which cancer-associated signalling pathways dominated in our three rectal subtypes. To examine the potential cancer-related pathways within our subtypes, we investigated pathway-response signatures using PROGENy. PROGENy calculates an activity score for 14 cancer-related pathways based on gene expressions. Results suggested that TGFβ, NFκB, TNFα, JAK-STAT, hypoxia, and oestrogen pathways were activated in RSS2, whereas MAPK, WNT and EGFR pathways were inactivated in RSS3 (Fig. 4b and Supplementary Fig. 5A). We observed low TRAIL signalling, p53 and androgen enrichment in RSS1. Moreover, GSVA revealed low expression of the immune response, angiogenesis, inflammatory response, EMT and KRAS signalling pathways in RSS1 (Fig. 4c and Supplementary Fig. 5A). We also observed high activation of MYC targets, cell cycle, G2M checkpoint, translation and proliferation on colonic crypt gene sets in RSS1. High expressions in EPHB2 and LGR5 signature cancer stem cells and low expressions on late transit amplifying colonic crypt genes were detected in RSS2. GSVA also showed high angiogenesis and EMT activity and low fatty acid degradation in RSS2, and high expression of fatty acid degradation and late transit amplifying genes in RSS3 (Fig. 4c). Since RSS2 carries CMS4 characteristics and appeared to be a subgroup within CMS4, we also analyzed the molecular differences between CMS4/RSS2 tumours and CMS4/non-RSS2 tumours. Our results suggested that the RSS2 subgroup has higher cytotoxic lymphocytes, monocytic lineage, myeloid dendritic cells, endothelial cells and fibroblasts. Moreover, WNT, TRAIL, TNFα, TGFβ, p53, NFκB, estrogen, androgen, hypoxia and Jak-STAT pathways were all expressed more in RSS2 (Supplementary Fig. 5B)

Master regulator analysis employing the GeneXplain platform identified 26 unique master regulators (MR) in RSS1. A total of 91 MR genes were identified in RSS2, but none were identified in RSS3. Additionally, we found master regulator molecules associated with subtypes. We observed 62 unique MR molecules in RSS1, 125 in RSS2 and none in RSS3. Unique master regulatory genes in RSS2 included MMP, ITGA, ITGB, CCL, and TGF-β gene families. The most important master molecules in RSS2 were related to cell surface receptors and cytokines. IL1A, FPR1, FCGR3A, PTGS2, MMP7, CSF3 and DUSP26 were found to be the top master molecules in RSS2. Other unique master molecules for RSS2 included fibronectin, periostin, glutamate, formyl peptide receptor, chemokine and metalloproteinases families. 27 MR genes and 36 MR molecules were found in all three subtypes. (Supplementary Table 5).

#### Interrogation of RSS subtypes by multiplex analysis

**Fig. 5:**
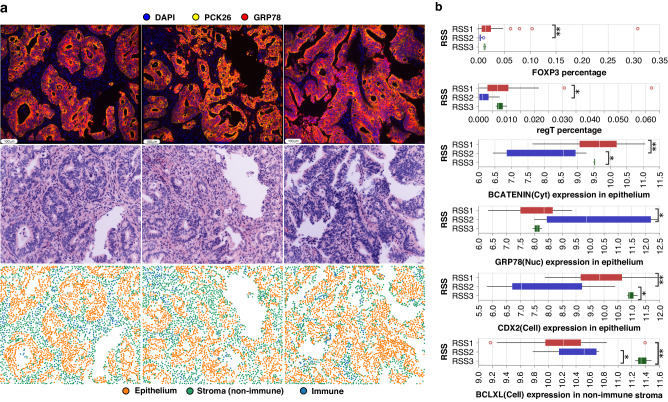
Multiplexed single-cell imaging analysis of RSS. **a** Highly multiplexed images (first row), virtual H&E images (second row), and cell type identifications (third row) of one of the RSS2 cancer tumours. All three TMAs belong to one patient, classified as RSS2. Colours of the multiplexed image: Blue-DAPI, Yellow-PCK26, and Red-GRP78. High GRP78 expression can be seen in epithelium tissues. **b** Boxplots of the immune cell percentages (first row) and expression levels of GRP78, β-catenin, CDX2 and Bcl-xl markers (Second and third row respectively). High expression of CDX2 in RSS3, GRP78 in RSS2, and low expression of β-catenin and Bcl-xl in RSS2 are observed at the single-cell level. Supporting the transcriptomics findings, higher Tregs infiltration is found in RSS1 and lower in RSS2. *p*-value annotations are as follows “**”*p* < 0.01; “*”*p* < 0.05; “ns” *p* > 0.05.

Lastly, we explored biological signatures in RSSs using tissue microarrays that were processed with CELL DIVE multiplexed immunofluorescence imaging for 56 markers of cell identity and cell signalling (Supplementary Table 4 shows the 56 markers used). 162,296 cells from 52 TMA cores and 18 pre-treatment naïve rectal cancer patients (14-RSS1, 3-RSS2, 1-RSS3) were analysed. We identified lower β-catenin expression in cancer cells specifically in RSS2 (Fig. 5). Cancer cells also showed higher levels of the ER stress marker GRP78 and lower levels of CDX2 in the RSS2 group. Bcl-xL expression was significantly higher in non-immune stromal cells in RSS3. The number of Tregs was found to be lower in RSS1, corroborating findings in the discovery/exploration cohort (Fig. 4). Representative virtual H&E images are also shown for the three subtypes.

## Discussion

Molecular subtyping can potentially deliver new prognostic tools and may direct treatment decisions. Here, we developed and validated a new subtyping method specifically for rectal cancers, focusing on the subtyping of pretreatment-naïve tissue samples, which can be readily determined from biopsies or non-pretreated resection tissue. Our new subtyping method developed, RSS subtyping, showed promising prognostic importance, where RSS1 showed better disease-free survival and RSS2 was associated with poorer DFS overall and in neoadjuvant-treated rectal cancers. Transcriptomic signatures of these subtypes associated RSS1 with low immune response and angiogenesis. Single-cell multiplexed imaging analysis revealed RSS2 had high stromal content and low FOXP3+ Tregs infiltration in the tumour. The most prominent signatures for RSS3 were microsatellite instability, a low CD4 + /CD8+ ratio along with the deactivation of MAPK, EGFR and WNT pathways.

Several studies in recent years suggested that colon and rectal cancers should be considered two different entities [31, 32]. Therefore, we started our analysis by determining molecular differences between colon and rectal cancer. Our results indicated there were several genes expressed differently between colon and rectal cancers, both in RNA-Seq and microarray studies. HOXB13, HOXC6 and CLDN8 were among the top DEGs. These findings supported previous studies that identified differentially expressed genes between the colon and rectal samples [33, 34]. Homeobox B13 (HOXB13) inhibits immune cell proliferation and promotes apoptosis through multiple pathways [35]. Specifically, downregulation of the HOXB13 gene was reported in right-sided colon cancers and higher expression is associated with poor prognosis. Furthermore, upregulation of HOXC6 was observed in right-sided colon cancers and lower expression in left-sided colons and rectal cancers was associated with poor prognosis [34]. HOX genes were also connected to carcinogenesis in colorectal cancer and studied as prognostic markers [36]. Similarly, CLDN8 was also associated with colorectal cancer prognosis [37]. These findings suggest that the differences between rectal cancers and colon cancers at the molecular level are important for prognosis prediction and can be crucial to identify treatment responses to neoadjuvant therapies.

To demonstrate the effect of neoadjuvant therapy on CMS subtyping in rectal cancer, we tested for subtyping differences between matched biopsy and resection samples in rectal cancer patients who were treated with neoadjuvant CRT. We demonstrated that CMS subtype distribution pre- and post-treatment were significantly different in these patients. More than half of the rectal resection samples were classified as CMS4. CMS4 was identified as a mesenchymal subtype with a high density of stroma and immune cells, poor survival and therapy resistance. Therefore, the CMS ‘switch’ we observed among patients’ findings could suggest that mostly therapy-resistant parts of the tumour were left after neoadjuvant treatment, or that profound tissue remodelling occurred. We also observed a fibroblast gene expression increase which might imply that CRT might be causing a wound-healing fibrotic phenotype in rectal tumours. Such changes are not unique to rectal cancers, for example, several studies regarding glioblastoma multiforme reported that chemo(radio)therapeutic agents cause a proneural to mesenchymal transition [38, 39]. Our findings suggest that a similar transition might be occurring in rectal cancers. Indeed, multiple studies showed how gene expression profiles of tumours are altered after CRT [5, 40]. We cannot fully exclude that some of the differences between pre-treatment biopsies and post-operative resections may be due to differences in sampling methods as a confounding factor. However, the strong changes in molecular subtypes we observed argue that prior exposure to CRT should be taken into consideration when identifying molecular subtypes in rectal cancer.

We, therefore, focused our rectal subtyping on pretreatment-naïve rectal samples. We showed that existing subtyping methods (CMS and CRIS subtyping) have different prognostic potentials in rectal cancers compared to colon cancers. In direct comparison, we observed that rectal cancers had better disease-free survival in tumours classified as CMS4, CRIS-B and CRIS-E, and colon cancers had a better prognosis in CMS3 and CRIS-A subtypes. Our findings also confirmed that within the group of colon cancers, the CMS4 subtype showed worse disease-free survival similar to previous studies [7]. However, when focused on rectal samples, we observed CMS4 and CMS3 had worse DFS than CMS2 but the multilevel model showed no significant difference between all CMS groups. One possible reason CMS has different and less significant prognostic patterns among rectal cancers is that more than 85% of the samples used in the CMS subtyping study were colon samples. The potential over-representation of colon samples in these models along with biological differences between the tumours and differences in treatment settings might be the reasons for differences in prognostic patterns of CMS/CRIS subtyping in rectal samples.

Because of the differences in the prognosis of CMS or CRIS subtypes in colon and rectal cancers, we performed a separate subtyping effort and identified and validated three rectal cancer-specific subtypes (RSS1-3). The training dataset had a good balance of treatment-naïve resection samples (51.6%) and pre-treatment biopsies (48.4%). Similar to CMS and CRIS, we also classified RSS subtypes using gene modules. This method helps researchers to identify subtypes even with a limited number of gene expressions. Since RSS was trained on microarray rectal samples with a balanced resection-biopsy ratio, it can be used in both types of samples. Of note, we demonstrated that RSS subtypes offer important prognostic value for rectal cancers. Our results showed that RSS2 had worse, and RSS1 best disease-free survival (Fig. 3f).

We also identified that RSSs had distinct molecular features and disease pathways activated. RSS2 had high stromal infiltration and activation of TGFβ, NFκB, and TNFα pathways. These results suggest RSS2 and CMS4 show significant resemblance. 62% of the CMS4 samples are classified as RSS2 and 99% of the RSS2 samples are classified as CMS4. This indicates that RSS2 can be described as a subgroup within CMS4. Moreover, we analysed the prognostic patterns between samples that identified both CMS4 and RSS2, and CMS4 and non-RSS2 samples. Our results showed the RSS2/CMS4 group to have a poorer prognosis than other groups, although the statistical significance was not reached due to the small sample size (Supplementary Fig. 6, *p*-value: 0.09). Biological characteristics of RSS2/CMS4 compared to non-RSS2/CMS4 also showed that the main characteristics of CMS4, such as high fibroblasts and endothelial cells, are more prominent in the RSS2 subgroup (Supplementary Fig. 5). This result suggests that RSS2 can be a more reliable subgroup for prognosis within CMS4 for rectal cancers.

RSS2 displayed higher expression of cancer stem cell markers, angiogenesis, TGF-β, and WNT signalling pathway activity (Fig. 4). These findings were also supported by master regulator analysis where periostin was shown as a unique master regulator for RSS2. Higher levels of periostin instigate angiogenesis, enhance WNT signalling and result in a microenvironment propitious for tumour progression and metastasis [41]. Periostin also contributes to cancer stem cell or mesenchymal stem cell attributes in the colorectal mucosa and helps to sustain stemness, which correlates with poor chemotherapy response [42]. Our results regarding EPHB2 and LGR5 stem-cell signatures support the importance of periostin for cancer stem-cell niches at the pericryptal regions for tumour development. Concerning the immune signatures of the RSS, both transcriptomics and single-cell multiplexed imaging analysis confirmed that RSS2 has lower Tregs infiltration in the tumour. More specifically, we identified low FOXP3+ infiltration in RSS2. Tregs are involved in suppressing immune responses through multiple processes and possibly affect the poor therapy response of this group. Master regulator analyses also showed immunosuppressive cytokines and chemokines such as CCL28, TGF-β and interleukin molecules to be among the unique master regulators for RSS2. It is reported that Tregs are involved in the production of these cytokines and chemokines [43]. Tumours with high Tregs were often associated with poor prognosis in many cancers, however, in colorectal cancer contrary to others the high abundance of FOXP3+ Tregs was associated with a good prognosis [44, 45]. Our results regarding RSS2 in rectal tumours support the concept that low FOXP3+ Treg infiltration in tumours is associated with poor outcomes in colorectal cancers. RSS3 was found to be enriched for microsatellite instability. Furthermore, this group exhibited high CD8 + T cell, low CD4 + T cell activity and low neutrophils as well as deactivation of MAPK, WNT and EGFR pathways. Pathway and gene set enrichment analyses revealed that RSS1 exhibits a low immune score and high tumour purity. The low expression of angiogenesis and inflammatory response genes in RSS1 was in line with a better prognosis. Considering the good prognosis of this group, it can be described as the group that benefits best from the treatments.

In conclusion, we have found that CMS and CRIS classifications convey different prognostic information in rectal versus colon cancers and that neoadjuvant therapy affects molecular subtypes in rectal cancers. Therefore, we developed RSS derived from treatment-naïve samples which showed promising prognostic results compared to the existing colorectal molecular subtypes. Our findings on the biological characterisation of these subtypes identify RSS1 as a low immune response group with a good prognosis, RSS2 as a high stromal and immune infiltration group with a poor prognosis, and RSS3 as MSI, low CD4+ and high CD8 + T cell activated group.

### Supplementary information

Supplementary Figures and Tables

## Data Availability

Public microarray datasets used in this study can be accessed at the Gene Omnibus website with the following IDs: GSE12945, GSE14333, GSE17536, GSE18088, GSE35452, GSE37892, GSE39084, GSE39582, GSE41258, GSE45404, GSE56699, GSE94104, GSE68204, GSE87211, GSE46862, GSE3493, GSE15781, GSE233517, and TCGA-COAD-READ. CellDive multiplexed images generated and analysed during the current study are available from the corresponding author upon reasonable request.

## Code availability

Codes for the RSS classifier are written in Python (version 3.8.10) and can be found at: github.com/kisakol/RSS_Classifier.
