# Supplementary material for: Identification of unique rectal cancer-specific subtypes
Source: Br J Cancer. 2024 Mar 26;130(11):1809–18. doi: 10.1038/s41416-024-02656-0 (PMC11130168; doi:10.1038/s41416-024-02656-0)
Supplement: Supplementary file 1 — Supplementary Figures and Tables [file 41416_2024_2656_MOESM1_ESM.pdf]

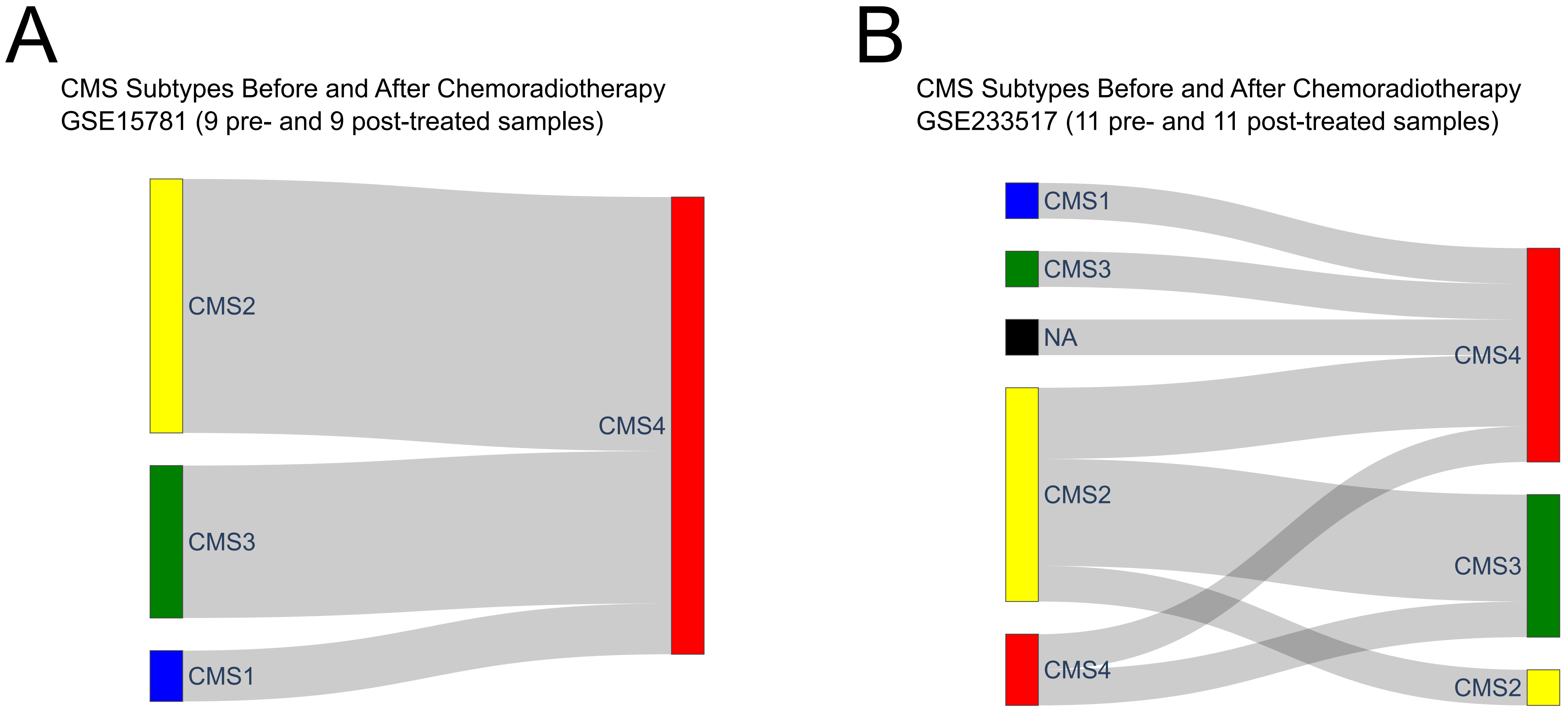

Supplementary Figure 1. Sankey Graphs of subtype changes after Chemoradiotherapy of the Two Additional Rectal Datasets. 9 matching pre- and post-treatment rectal samples from GSE15781 (A) and 11 matching samples from GSE233517 (B) datasets. All the datasets indicate there is a huge shift to CMS4 after chemo(radio)therapy.

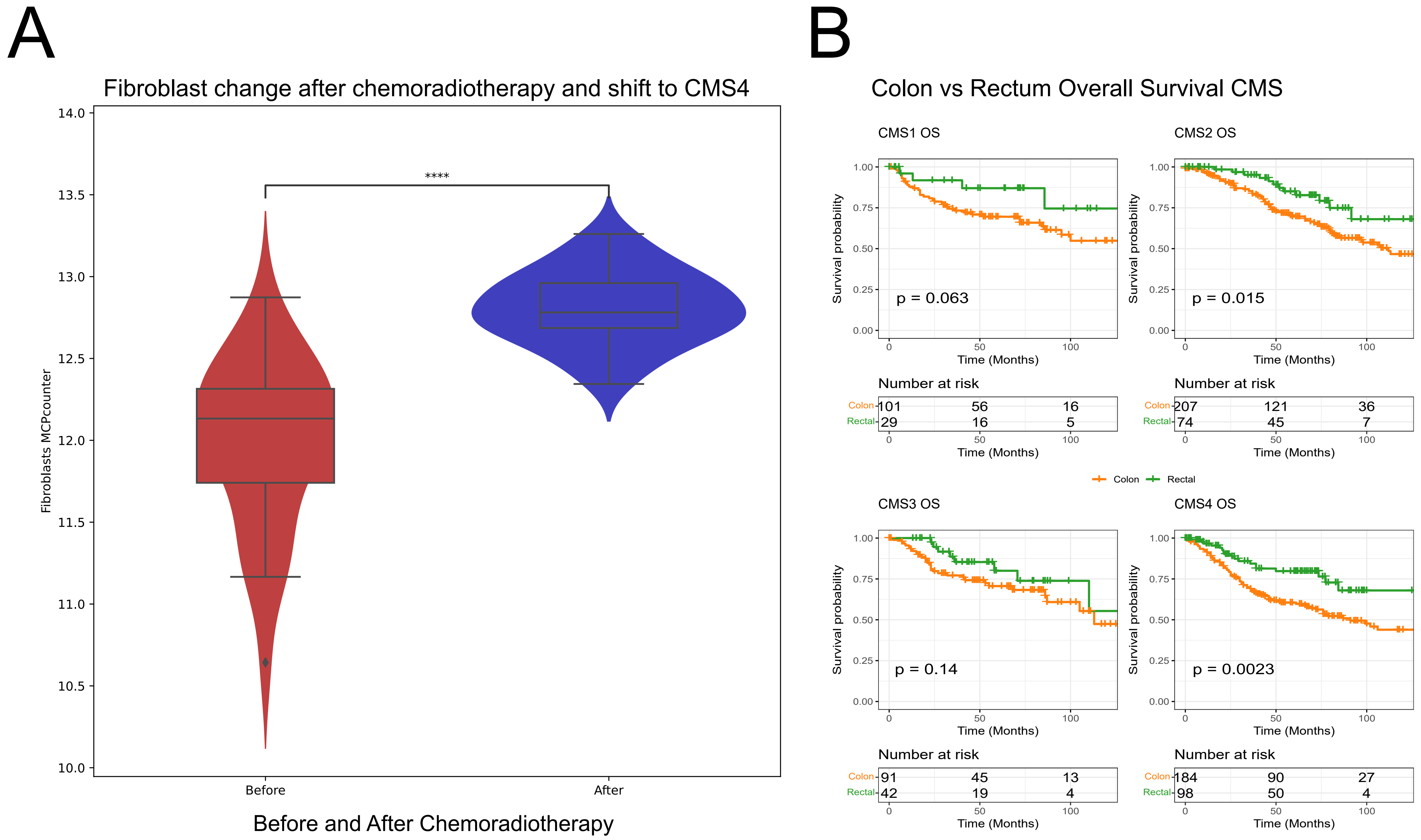

Supplementary Figure 2. A- Boxplot represents high fibroblast enrichment of CMS4 samples from GSE94104 study. Samples classified as CMS4 after chemoradiotherapy are selected (CMS classifications before the chemoradiotherapy can be seen in Figure 2A). B- Kaplan-Meier plots show there is a significant difference between colon and rectum cancers in overal survival of CMS4 and CMS2 tumours.

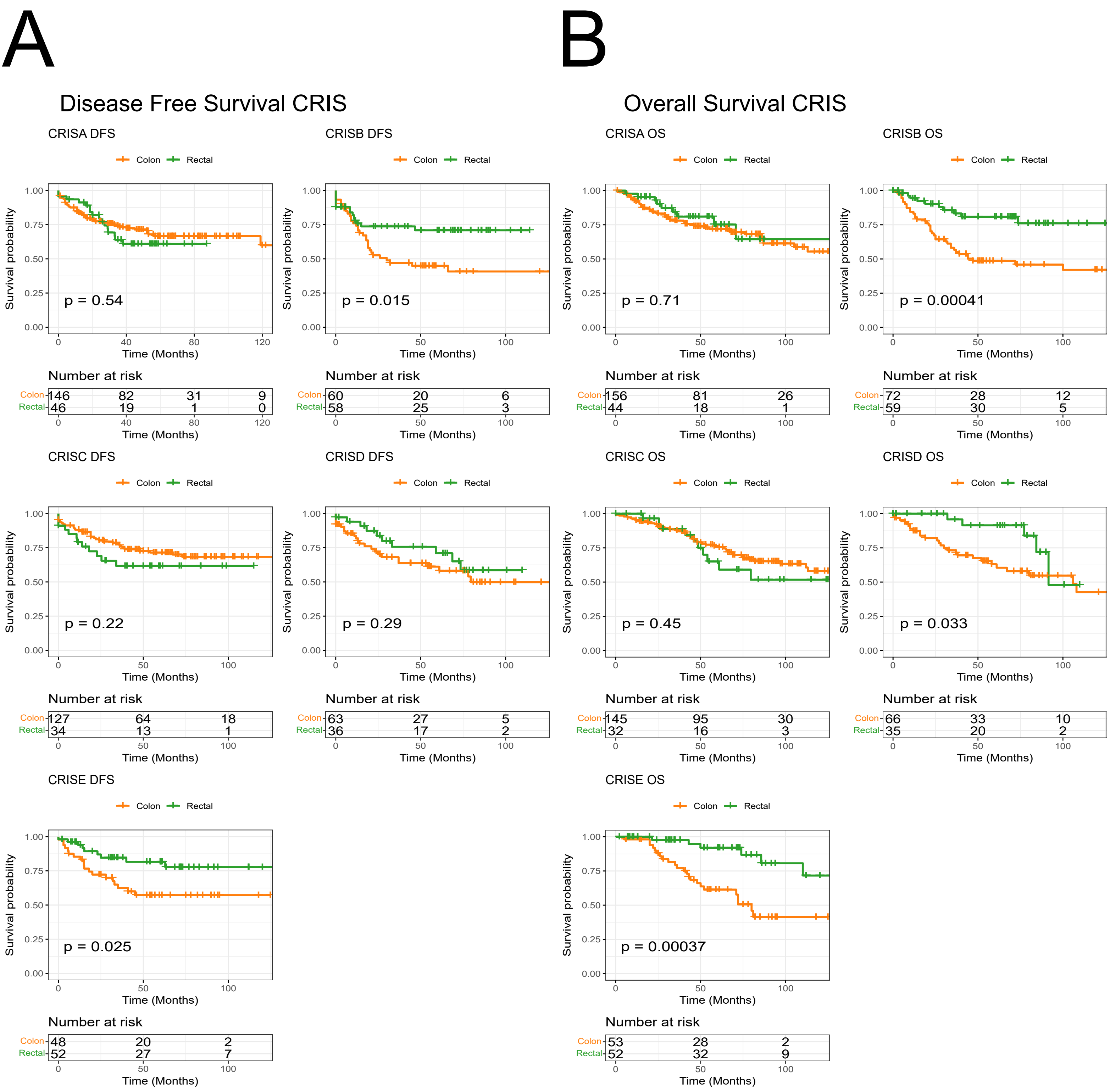

Supplementary Figure 3. (A) Kaplan-Meier plots of CRIS shows significant prognostic differences between colon and rectum cancers in CRIS-B and CRIS-E subtypes where rectal tumours have better DFS. (B) When focused on overall survival we observed CRIS-B/D/E subtypes has worse prognosis on colon cancers compared to rectal cancers.

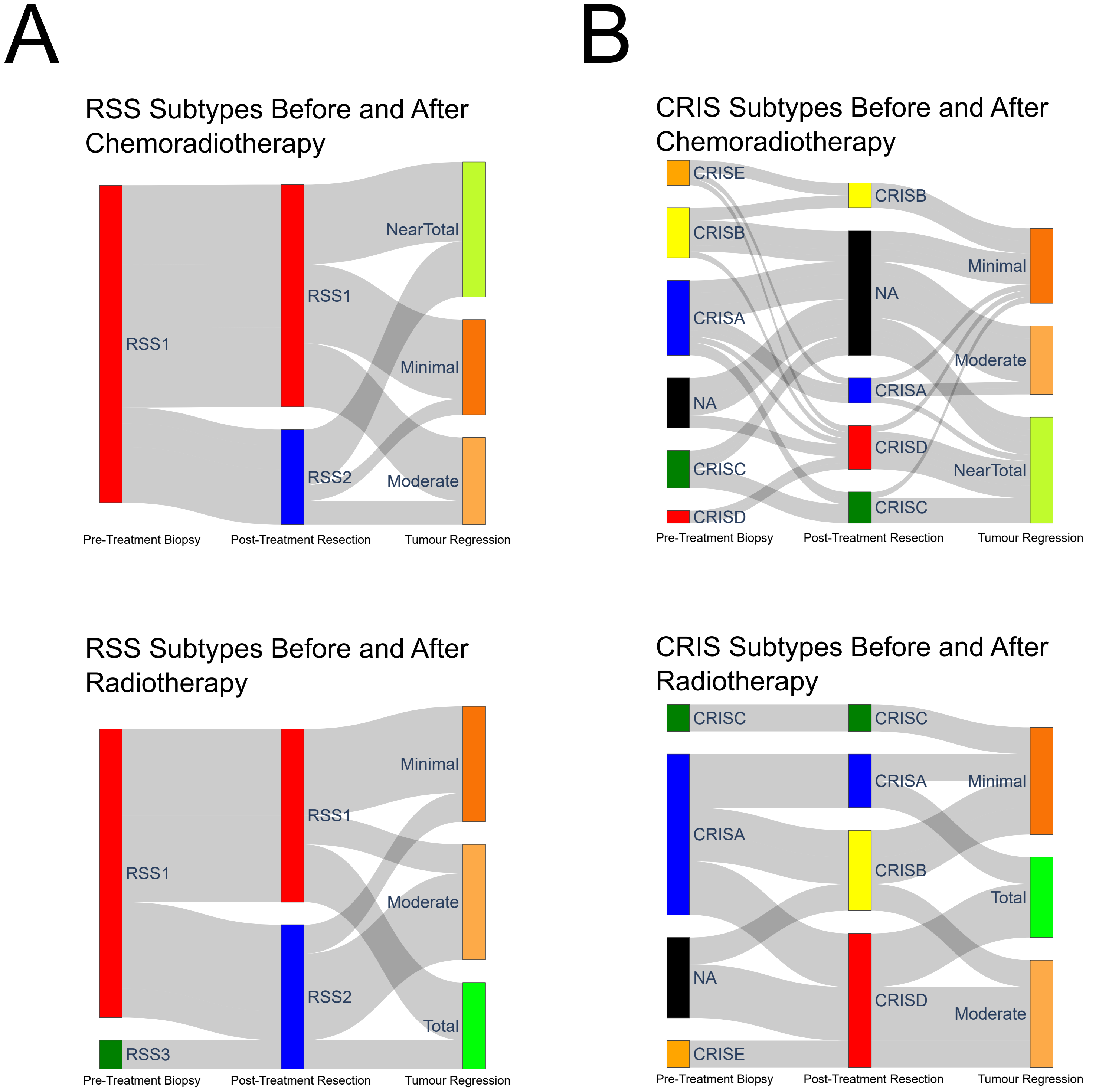

Supplementary Figure 4. Sankey Graphs of subtype changes after Chemoradiotherapy and radiotherapy. Matching samples from GSE94101 (upper Sankey graphs) and GSE56699 (lower Sankey graphs) datasets are used. A-Rectal Specific Subtype changes after radiotherapy and chemoradiotherapy show that there is a significant shift to RSS2 after therapy. B- CRIS subtype changes after radiotherapy show a transition to CRISD subtype and after chemoradiotherapy reveal a transition to unclassified or mixed subtypes.

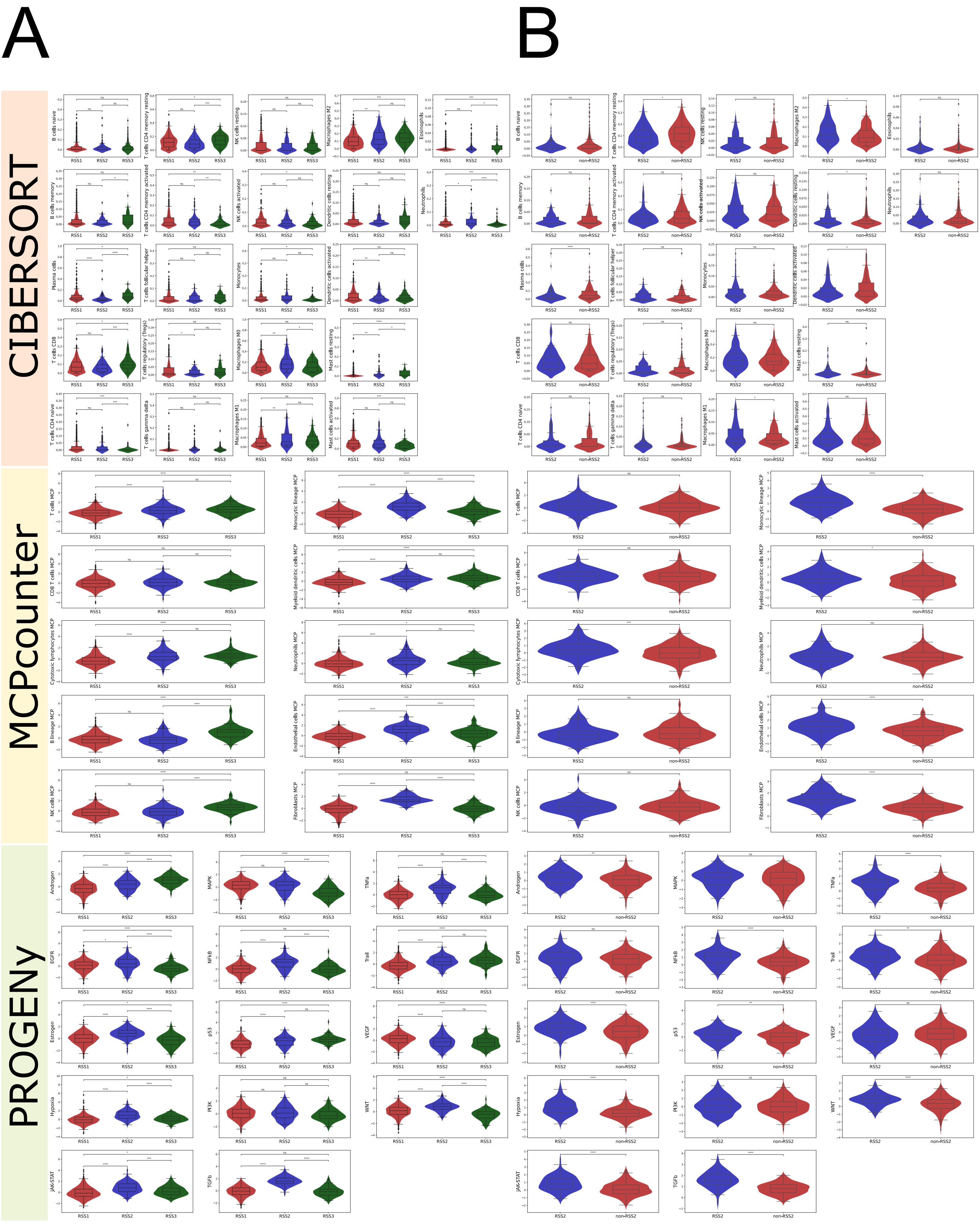

Supplementary Figure 5. Boxplots of immune signatures from CIBERSORT and MCPcounter methods and cancer pathway activity scores from PROGENy are presented. A- Boxplots represents the differences between Rectal Specific Subtypes. B- Boxplots show the differences between CMS4/RSS2 and CMS4/non-RSS2 classes.

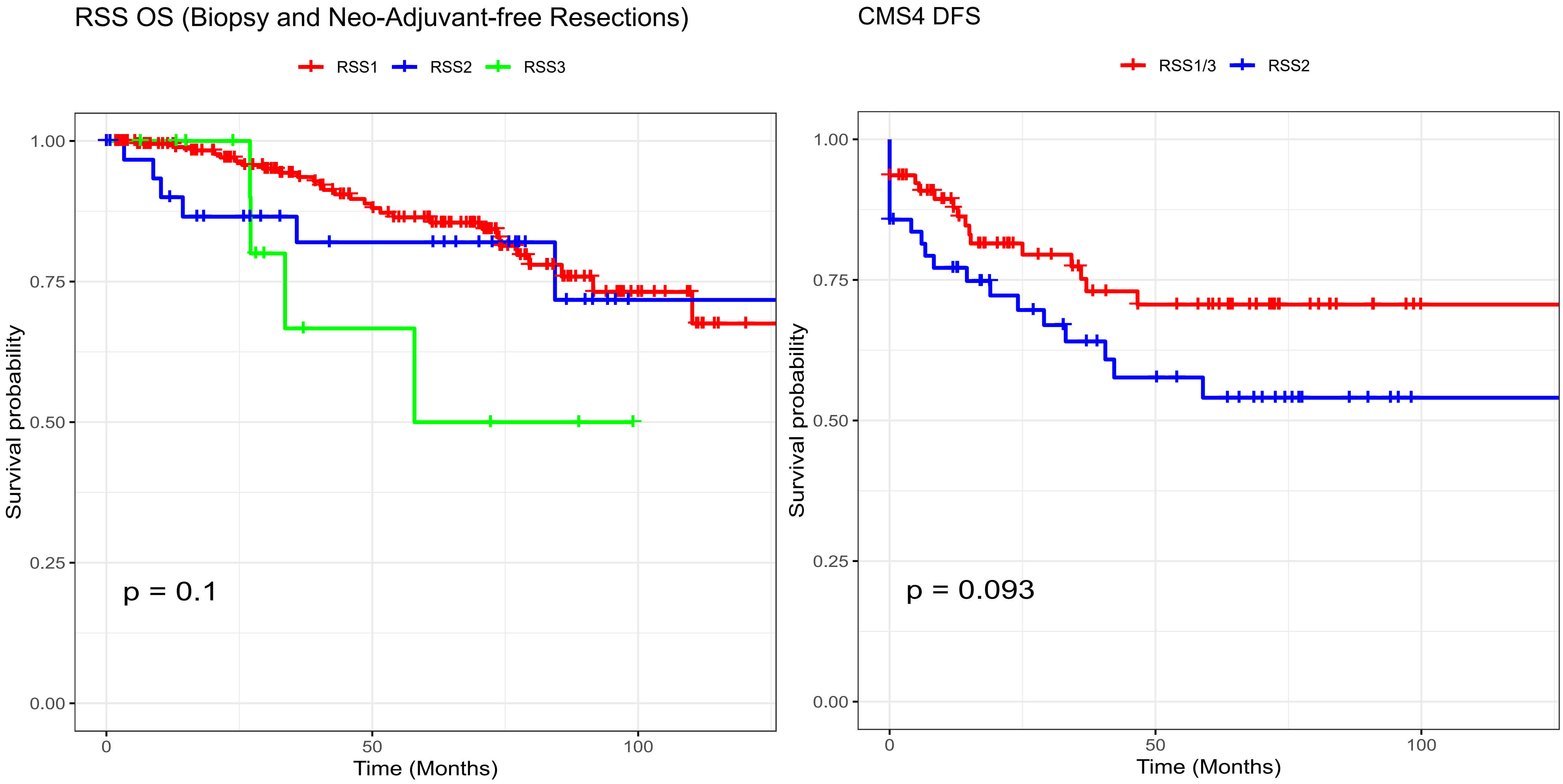

Supplementary Figure 6. Kaplan Meier plots show the overall survival patterns of rectal specific subtypes (left), and CMS4/RSS2 vs CMS4/non-RSS2.

Datasets	Sample Size N (Colon, Rectal)	Platform ID	Platform
GSE12945	62 (29, 33)	GPL96	Affymetrix Human Genome U133A Array
GSE14333	289 (250, 39)	GPL570	Affymetrix Human Genome U133 Plus 2.0 Array
GSE17536	11 (11, 0)	GPL570	Affymetrix Human Genome U133 Plus 2.0 Array
GSE18088	53 (53, 0)	GPL570	Affymetrix Human Genome U133 Plus 2.0 Array
GSE35452	46 (0, 46)	GPL570	Affymetrix Human Genome U133 Plus 2.0 Array
GSE37892	47 (47, 0)	GPL570	Affymetrix Human Genome U133 Plus 2.0 Array
GSE39084	70 (62, 8)	GPL570	Affymetrix Human Genome U133 Plus 2.0 Array
GSE39582	505 (505, 0)	GPL570	Affymetrix Human Genome U133 Plus 2.0 Array
GSE41258	166 (152, 14)	GPL96	Affymetrix Human Genome U133A Array
GSE45404	42 (0, 42)	GPL570	Affymetrix Human Genome U133 Plus 2.0 Array
Total	1291 (1109, 182)		

Supplementary Table 1A. 10 Microarray datasets were processed with RMA normalization and applied batch effect correction. These datasets were used to differentiate colon vs rectum differences. 182 rectal samples were also used as a training/discovery dataset for the rectal-specific clustering. Duplicate entries in the GSE39582, GSE37892, GSE39084, GSE17536 and GSE14333 studies were detected with comparing md5 checksums of the RAW files. Duplicate entries were removed from further analyses and only one entry for each kept. Therefore, the number of samples in these studies were reduced compared to the original entries at GEO database.

Datasets	Sample Size N (Colon, Rectal)	Platform ID	Platform
GSE68204	59 (0, 59)	GPL6480	Agilent-014850 Whole Human Genome Microarray 4x44K G4112F
GSE87211	203 (0, 203)	GPL13497	Agilent-026652 Whole Human Genome Microarray 4x44K v2
GSE46862	69 (0, 69)	GPL6244	Affymetrix Human Gene 1.0 ST Array
GSE3493	46 (0, 46)	GPL8300	Affymetrix Human Genome U95 Version 2 Array
Total	377 (0, 377)		

Supplementary Table 1B. First cohort of validation/exploration rectal microarray datasets. Processed and normalized gene expression versions of these datasets were used for GSVA/PROGENy/Immune activity analyses and subtyping classification. These analyses and classifications were carried out separately for each dataset.

Datasets	Sample Size N (Colon, Rectal)	Platform ID	Platform
GSE94104	80* (0, 80)	GPL14951	Illumina HumanHT-12 WG-DASL V4.0 R2 expression beadchip
GSE56699	72** (0, 72)	GPL14951	Illumina HumanHT-12 WG-DASL V4.0 R2 expression beadchip
GSE15781	18*** (0, 18)	GPL2986	ABI Human Genome Survey Microarray Version 2
GSE233517	22*** (0, 22)	GPL18573	Illumina NextSeq 500
Total	192 (0, 192)		

Supplementary Table 1C. Second cohort of validation/exploration rectal microarray datasets. These datasets contain pre-treatment and post-treatment matching samples from the same patients. Therefore, they were used to demonstrate neoadjuvant effect on subtyping but excluded from survival analyses due to potential multiple assignments of subtypes to the same patients.

* GSE94104 has 40 biopsy and 40 resection samples from same patients, total number of patients = 40. No survival data, therefore excluded from survival analysis but used in GSVA, PROGENy, Immune cell activity analyses.

** GSE56699 dataset has 11 biopsy and 11 resection samples from same patients but also contains multiple biopsy or resection samples or only biopsy/resection samples. Total Number of Patients = 52. Classifications from biopsy samples use for survival analysis. Also used in GSVA, PROGENy, Immune cell activity analyses.

*** GSE233517 has 11 biopsy and 11 resection samples from same patients, total number of patients = 22. This dataset was only used to demonstrate the effect of subtyping with neoadjuvant therapy and is not included in other analyses. Due to the small number size and the difference in their sequencing platform compared to the rest.

*** GSE15781 has 9 biopsy and 9 resection samples from same patients, total number of patients = 18. This dataset was

only used to demonstrate the effect of subtyping with neoadjuvant therapy and is not included in other analyses. Due to the small number size and the difference in their sequencing platform compared to the rest.

Datasets	Sample Size N (Colon, Rectal)	Platform ID	Platform
TCGA-COAD-READ	608 (449, 159)	IlluminaHiSeq_RNASeq	Illumina HiSeq 2000 RNA Sequencing
Total	608 (449, 159)		

Supplementary Table 1D. TCGA-COAD-READ dataset was downloaded from https://gdc.cancer.gov/about-data/publications/pancanatlas

	Colon (N=1109)	Rectal (N=182)	Total (N=1291)	p value
**Sex**				0.552
N-Miss	0	46	46	
Female	511 (46.1%)	59 (43.4%)	570 (45.8%)	
Male	598 (53.9%)	77 (56.6%)	675 (54.2%)	
**Age**				0.003
N-Miss	1	46	47	
Mean (SD)	65.980 (13.152)	62.390 (12.570)	65.588 (13.132)	
Range	19.000 - 97.000	20.000 - 87.000	19.000 - 97.000	
**Procedure**				< 0.001
Biopsy	0 (0.0%)	88 (48.4%)	88 (6.8%)	
Resection	1109 (100.0%)	94 (51.6%)	1203 (93.2%)	
**T Stage**				< 0.001
N-Miss	328	127	455	
0	1 (0.1%)	0 (0.0%)	1 (0.1%)	
1	14 (1.8%)	0 (0.0%)	14 (1.7%)	
2	74 (9.5%)	19 (34.5%)	93 (11.1%)	
3	546 (69.9%)	35 (63.6%)	581 (69.5%)	
4	143 (18.3%)	1 (1.8%)	144 (17.2%)	
Tis	3 (0.4%)	0 (0.0%)	3 (0.4%)	
**N Stage**				0.635
N-Miss	387	127	514	
0	397 (55.0%)	33 (60.0%)	430 (55.3%)	
1	178 (24.7%)	10 (18.2%)	188 (24.2%)	
2	141 (19.5%)	12 (21.8%)	153 (19.7%)	
3	6 (0.8%)	0 (0.0%)	6 (0.8%)	
**M Stage**				0.290
N-Miss	534	139	673	
0	397 (69.0%)	33 (76.7%)	430 (69.6%)	
1	178 (31.0%)	10 (23.3%)	188 (30.4%)	
**Adjuvant Therapy**				< 0.001
N-Miss	240	143	383	
Chemoradiotherapy	18 (2.1%)	7 (17.9%)	25 (2.8%)	
Chemotherapy	320 (36.8%)	4 (10.3%)	324 (35.7%)	
No	530 (61.0%)	28 (71.8%)	558 (61.5%)	
Radiotherapy	1 (0.1%)	0 (0.0%)	1 (0.1%)	
**Neoadjuvant Therapy**				< 0.001
N-Miss	797	47	844	
Chemoradiotherapy	0 (0.0%)	88 (65.2%)	88 (19.7%)	
No	312 (100.0%)	47 (34.8%)	359 (80.3%)	
**CMS**				0.021
N-Miss	128	35	163	
CMS1	180 (18.3%)	13 (8.8%)	193 (17.1%)	
CMS2	321 (32.7%)	48 (32.7%)	369 (32.7%)	
CMS3	169 (17.2%)	34 (23.1%)	203 (18.0%)	
CMS4	311 (31.7%)	52 (35.4%)	363 (32.2%)	
**CRIS**				0.722
N-Miss	269	47	316	
CRISA	274 (32.6%)	43 (31.9%)	317 (32.5%)	
CRISB	128 (15.2%)	19 (14.1%)	147 (15.1%)	
CRISC	242 (28.8%)	39 (28.9%)	281 (28.8%)	
CRISD	110 (13.1%)	15 (11.1%)	125 (12.8%)	
CRISE	86 (10.2%)	19 (14.1%)	105 (10.8%)	

	Colon (N=1109)	Rectal (N=182)	Total (N=1291)	p value
**KRAS**				0.556
N-Miss	563	174	737	
M	217 (39.7%)	4 (50.0%)	221 (39.9%)	
WT	329 (60.3%)	4 (50.0%)	333 (60.1%)	
**BRAF**				0.348
N-Miss	596	174	770	
M	51 (9.9%)	0 (0.0%)	51 (9.8%)	
WT	462 (90.1%)	8 (100.0%)	470 (90.2%)	
**DFS (Months)**				0.633
N-Miss	253	109	362	
Mean (SD)	44.832 (37.207)	42.703 (27.902)	44.664 (36.554)	
Range	0.000 - 201.000	0.000 - 118.580	0.000 - 201.000	
**DSS (Months)**				0.010
N-Miss	878	166	1044	
Mean (SD)	47.698 (31.204)	68.883 (36.719)	49.070 (31.936)	
Range	0.920 - 142.550	2.360 - 134.860	0.920 - 142.550	
**OS (Months)**				0.382
N-Miss	149	119	268	
Mean (SD)	55.980 (38.299)	60.335 (38.568)	56.248 (38.311)	
Range	0.000 - 203.000	2.360 - 196.000	0.000 - 203.000	

Supplementary Table 2A. Clinical information of the training datasets shown in Supplementary Table 1A

	RSS1 (N=688)	RSS2 (N=119)	RSS3 (N=63)	Total (N=870)	p value
**Sex**					0.479
N-Miss	200	32	12	244	
Female	183 (37.5%)	34 (39.1%)	15 (29.4%)	232 (37.1%)	
Male	305 (62.5%)	53 (60.9%)	36 (70.6%)	394 (62.9%)	
**Age**					0.042
N-Miss	201	32	12	245	
Mean (SD)	62.881 (11.282)	59.590 (11.297)	62.827 (10.943)	62.418 (11.296)	
Range	29.600 - 90.000	20.000 - 83.000	38.000 - 87.000	20.000 - 90.000	
**Stage**					0.010
N-Miss	455	75	42	572	
1	48 (20.6%)	7 (15.9%)	3 (14.3%)	58 (19.5%)	
2	69 (29.6%)	16 (36.4%)	13 (61.9%)	98 (32.9%)	
3	88 (37.8%)	10 (22.7%)	4 (19.0%)	102 (34.2%)	
4	28 (12.0%)	11 (25.0%)	1 (4.8%)	40 (13.4%)	
**Grade**					0.283
N-Miss	660	113	56	829	
Moderately Differentiated	15 (53.6%)	4 (66.7%)	6 (85.7%)	25 (61.0%)	
Poorly Differentiated	13 (46.4%)	2 (33.3%)	1 (14.3%)	16 (39.0%)	
**CMS**					< 0.001
N-Miss	148	0	3	151	
CMS1	66 (12.2%)	1 (0.8%)	9 (15.0%)	76 (10.6%)	
CMS2	214 (39.6%)	0 (0.0%)	3 (5.0%)	217 (30.2%)	
CMS3	97 (18.0%)	0 (0.0%)	33 (55.0%)	130 (18.1%)	
CMS4	163 (30.2%)	118 (99.2%)	15 (25.0%)	296 (41.2%)	
**CRIS**					< 0.001
N-Miss	175	37	1	213	
CRISA	114 (22.2%)	13 (15.9%)	46 (74.2%)	173 (26.3%)	
CRISB	92 (17.9%)	28 (34.1%)	4 (6.5%)	124 (18.9%)	
CRISC	121 (23.6%)	9 (11.0%)	9 (14.5%)	139 (21.2%)	
CRISD	81 (15.8%)	30 (36.6%)	1 (1.6%)	112 (17.0%)	
CRISE	105 (20.5%)	2 (2.4%)	2 (3.2%)	109 (16.6%)	
**DFS (Months)**					0.441
N-Miss	399	62	37	498	
Mean (SD)	44.680 (33.016)	41.687 (35.239)	36.712 (25.110)	43.664 (32.877)	
Range	0.000 - 151.170	0.000 - 152.480	0.000 - 99.033	0.000 - 152.480	
**DSS (Months)**					0.674
N-Miss	554	91	58	703	
Mean (SD)	27.471 (23.705)	29.483 (22.385)	36.440 (39.103)	28.077 (23.902)	
Range	0.000 - 131.067	1.033 - 98.167	2.533 - 99.033	0.000 - 131.067	
**OS (Months)**					0.738
N-Miss	311	50	38	399	
Mean (SD)	44.903 (34.307)	43.081 (32.937)	40.034 (25.293)	44.377 (33.655)	

	RSS1 (N=688)	RSS2 (N=119)	RSS3 (N=63)	Total (N=870)	p value
Range	0.000 - 196.000	0.000 - 152.480	2.533 - 99.033	0.000 - 196.000	

Supplementary Table 2B. Clinical information of the 870 rectal cancer samples

Analysis	Explanation of the Datasets Used
Colon vs Rectal DE	Training Cohort of 1291 colorectal samples (1109, 182) and 608 samples from TCGA-COAD-READ (449, 159) as a validation set.
Effect of neoadjuvant Chemo(radio)therapy	A total of 142 matching pre-treatment biopsy (n=71) and post-treatment resection (n=71) samples were analyzed to demonstrate the effect of neoadjuvant therapy on the molecular subtyping. GSE94104 (n=80), GSE56699 (n=22), GSE233517 (n=22), and GSE15781 (n=18). Figure 3A shows the results from GSE94104 (n=80), GSE56699 (n=22) and Supplementary Figure 1 shows the shift for GSE233517 (n=22) and GSE15781 (n=18) datasets.
Colon vs Rectal Disease-Free Survival Analysis with CMS	Training cohort and the first validation and cohorts were used for the survival analyses. The samples with unknown CMS/CRIS labels and missing disease-free survival or overall survival information were removed. Only the samples where subtyping information acquired from treatment naive samples were used. In the case of Figure 2B, A total of 770 treatment-naive samples with DFS information were available for CMS1/2/3/4 tumours. Other survival plots (Supplementary Figures 2-3-6) involve the risk table annotations which show the number of patients according to the same criteria described.
RSS Classification	182 Rectal samples from the training cohort are used for identifying the rectal-specific subtypes.
PROGENy, CIBERSORT, MCPcounter, and GSVA	A total of 870 Rectal Samples were used. Training Cohort (n=182) + TCGA (n = 159) + First Validation/Exploration Microarray Cohort (n=377) + Second Validation/Exploration Microarray Cohort (n=152, only GSE94104 (n=80) and GSE56699 (n=72))
Master Regulators	Only GSE87211 dataset was used with 203 tumour and 160 normal samples. RSSs were classified in tumour samples and each RSS group was compared to the normal samples using geneXplain platform.

Supplementary Table 3. Detailed explanations of samples used in different analyses.

Target	Clone	Vendor	Catalog
ALDH1	44	BD Bio	611195
Apaf-1	2E12	Millipore	MAB3503
ATP5A	EPR13030(B)	Abcam	ab196198
Bak	Y164	Abcam	ab220790
Bax	E63	Abcam	ab216985
BCL-2	E17	Abcam	ab190577
Bcl-xL	7D9	Thermo	MS-1334
beta-catenin	15B8	Sigma	C7738
Bid	B-3	Santa Cruz	sc-373939
Bim	polyclonal	R&D	AF1325
Caspase 3 cleaved	5A1E	Cell Signaling	9664
Caspase-3 (Pro+cleaved)	D3R6Y	Cell Signaling	14214
CD20	EP459Y	Abcam	ab198943 A647
CD3	F7.2.38	Dako	M7254
CD31	89C2	Cell Signaling	3528
CD34	QBEnd/10	Leica	END-L-CE
CD4	EPR6855	Abcam	ab181724
CD68	KP1	Thermo	MS-397
CD8	C8/144B	Dako	M7103
CDX2	D11D10	Cell Signaling	12306
cIAP-1	polyclonal	R&D	AF8181
C-Myc	Y69	Abcam	ab190560
Collagen IV	IV-4H12	Millipore	MAB3326
Cytokeratin AE1	AE1	eBioscience	14-9001
Cytokeratin PCK26	PCK26	Sigma	C1801
EPCAM	E144	Epitomics	1144
FADD	EPR5030	Abcam	ab229444
FLIP	896537	R&D	MAB8430
FLIP	D5J1E	Cell Signaling	56343
FOXP3	206D	Biolegend	320014
G6PD	G-12	Santa Cruz	sc-373886
GAPDH	14C10	Cell Signaling	3907
Glut-1	polyclonal	Millipore	07-1401
Granzyme B	GrB-7	Dako	M7235
GRP78	C50B12	Cell Signaling	3177
Hexokinase-II	C64G5	Cell Signaling	2867
HLA I	EMR8 5	Abcam	ab70328
HTR2B/SR2B	C-6	Santa Cruz	sc-376878 A647
Ki67	SP6	Zeta	Z2031
LDHA	C4B5	Cell Signaling	3582
MCL-1	C-2	Santa Cruz	sc-7443
MLKL1	3D4C6	Thermo	66675-1-Ig
MSH2	D24B5	Cell Signaling	2017
MSH6	EPR3945	Abcam	ab92471
MUC5	1-13M1	Abcam	ab24070

NAKATPase	EP1845Y	Abcam	ab167390
pan_Cadherin	polyclonal	Neomarkers	RB 9036 P
PD1	EPR4877(2)	Abcam	ab201825
PKM2	C6198	Cell Signaling	4053
Procaspase-8	EPR162	Abcam	ab108333
Procaspase-9	96.1.23	Santa Cruz	sc-56076 A647
RIP3	B-2	Santa Cruz	sc-374639
S6	C-8	Santa Cruz	sc-74459 A647
SDHA	B-1	Santa Cruz	sc-166909
SMA	1A4	Sigma	C6198
Smac	79-1-83	Cell Signaling	2954
TIGAR	polyclonal	R&D	R&D
XBP1	EPR4086	Abcam	ab109221
XIAP (API3)	polyclonal	Thermo	APH937
ZEB1	D80D3	Cell Signaling	3396

Supplementary Table 4. Sources of antibodies

RSS1 Master Molecules			
ID	Master molecule name	CMA rank	Total rank
MO000125815	FCgammaRIIIA(h)	17	28
MO000038335	fMLP-R:Gi:GDP	22	34
MO000036091	G-CSF(h)	8	39
MO000130281	G-CSF-isoform1(h)	8	40
MO000286201	G-CSF-isoform3(h)	8	40
MO000151288	G-CSF-isoform2(h)	8	42
MO000038336	fMLP:fMLP-R:Gi:GDP	22	45
MO000004274	E2F-1(h)	49	63
MO000021420	angiotensin II(h)	29	64
MO000019424	IL-6(h)	66	80
MO000036116	GM-CSF(h)	87	89
MO000032593	calcitonin(h)	89	93
MO000019977	MIP-3beta(h)	12	113
MO000135001	calcitonin-isoform2(h)	89	113
MO000205368	calcitonin-isoform3(h)	89	113
MO000205372	calcitonin-isoform1(h)	89	113
MO000125816	FCgammaRIIIA(h)	17	116
MO000089119	IL-11-isoform1(h)	97	117
MO000032592	BDNF(h)	70	120
MO000254403	BDNF-isoform3(h)	70	121
MO000254404	BDNF-isoform4(h)	70	121
MO000254405	BDNF-isoform5(h)	70	121
MO000254402	BDNF-isoform2(h)	70	124
MO000103495	BDNF-isoform1(h)	70	127
MO000033461	fMLP-R(h)	119	141
MO000281381	(angiotensin II)2:(AT2 receptor)2:(ATIP-isoform3)2:SHP-1	29	144
MO000281378	(angiotensin II)2:(AT2 receptor)2:(ATIP-isoform3)2	29	145
MO000280542	(angiotensin II)2:(AT2 receptor)2	29	146
MO000007287	inhibin beta A(h)	150	151
MO000035415	IL-2(h)	140	154
MO000161626	PC1-isoform1(h)	46	172
MO000161627	PC1(h)	46	172
MO000255342	FREM1-isoform2(h)	167	172
MO000286580	PC1-isoform2(h)	46	172
MO000220460	S100A12(h)	168	175
MO000102659	PGDS(h)	174	177
MO000031992	granzymeB(h)	117	186
MO000046121	Aurora-A{pT288}:tpx2{pS}	108	187
MO000046129	PP1:Aurora-A{pT288}:tpx2{pS}	108	187
MO000042660	tpx2(h){pS}	108	189
MO000114255	AMPKalpha-2(h)	77	189
MO000042658	tpx2(h):Aurora-A(h)	108	190
MO000042656	tpx2(h)	108	191
MO000046123	importin-alpha:importin-beta:tpx2:Kid	108	191
MO000109695	tpx2-isoform1(h)	108	191
MO000339227	tpx2-isoform2(h)	108	191
MO000004594	EphB1(h)	13	196
MO000137371	EphB1-isoform1(h)	13	204
MO000334533	EphB1-isoform2(h)	13	204
MO000334534	EphB1-isoform3(h)	13	204
MO000007322	IL-13(h)	191	212
MO000082741	IL-1alpha(h)	204	214
MO000019374	IL-1alpha(h)	204	218
MO000019852	I-TAC(h)	120	225
MO000221557	IBSP(h)	28	227
MO000018698	SS2R-isoform1(h)	133	230
MO000144599	SS2R-isoform2(h)	133	230
MO000144600	SS2R(h)	133	233
MO000125976	CD21-isoform-A(h)	61	235
MO000125982	CD21-isoform-C(h)	61	235
MO000125983	CD21-isoform-D(h)	61	235
MO000138687	GP39(h)	57	236
MO000125979	CD21-isoform-B(h)	61	238
MO000125978	CD21(h)	61	239
MO000070193	OSR1(h)	19	246
MO000080033	IL-6(h)	66	248
MO000059880	CD22(h)	101	254
MO000206060	CD22-alpha(h)	101	254
MO000286128	CD22-isoform3(h)	101	254
MO000311960	CD22-isoform4(h)	101	254
MO000327621	CD22-isoform5(h)	101	254
MO000025969	WT1 -KTS(h)	123	257
MO000025970	WT1 I(h)	123	257
MO000025971	WT1-del2(h)	123	257
MO000025972	WT1 I-del2(h)	123	257

MO000258120	WT1-isoform6(h)	123	257
MO000258121	WT1-isoform7(h)	123	257
MO000258122	WT1-isoform8(h)	123	257
MO000271719	WT1-isoform9(h)	123	257
MO000025253	WT1 I -KTS(h)	123	265
MO000102040	WT1(h)	123	266
MO000032678	trkC(h)	39	267
MO000059884	CD22(h){pY}	101	267
MO000114187	trkC-isoform1(h)	39	267
MO000256501	trkC-B(h)	39	267
MO000256502	trkC-C(h)	39	267
MO000256503	trkC-D(h)	39	267
MO000328032	trkC-isoform5(h)	39	267
MO000480287	trkC(h){p}	39	267
MO000480316	CD22(h){pY777}	101	267
MO000000322	G12/13	22	275
MO000180153	LOX-1-isoform1(h)	277	290
MO000207255	CHGA(h)	166	291
MO000089174	NF-E2 p45(h)	58	293
MO000004685	ERK4(h)	232	297
MO000023462	COX2(h)	283	297
MO000125307	MDA7(h)	292	298
MO000215287	MMP12(h)	100	302
MO000334232	M1-isoform2(h)	201	303
MO000060023	M1-isoform1(h)	201	304
MO000018765	M1(h)	201	305
MO000018285	Gq:GDP	22	313
MO000080123	GM-CSF(h)	87	313
MO000141169	lithostathine1alpha(h)	171	315
MO000320733	KLF4(h){ub}n	1	317
MO000322608	(angiotensin II)2:(AT1A)2:Gq:GDP	22	317
MO000125306	MDA7-isoform1(h)	292	319
MO000271183	MDA7-isoform2(h)	292	319
MO000271184	MDA7-isoform3(h)	292	319
MO000271185	MDA7-isoform4(h)	292	319
MO000281767	(angiotensin II)2:(AT1A{p})2:beta-arrestin2:Raf-1:MEK1:ERK2	29	321
MO000281768	(angiotensin II)2:(AT1A{p})2:beta-arrestin2:Raf-1:MEK1{pS218}{pS222}:ERK2{pT185}{pY187}	29	321
MO000255857	KLF4-isoform3(h)	1	323
MO000281766	(angiotensin II)2:(AT1A{p})2	29	323
MO000319000	KLF4-isoform4(h)	1	323
MO000319001	KLF4-isoform5(h)	1	323
MO000280543	(angiotensin II)2:(AT1A)2	29	324
MO000125555	KLF4-isoform1(h)	1	326
MO000125563	KLF4-isoform2(h)	1	327
MO000125561	KLF4(h)	1	328
MO000292758	(angiotensin II)2:(AT2 receptor)2:Cdk4:AMPKalpha-2:AMPKbeta-1{myr}:AMPKgamma-1:(AMP)3	29	331
MO000145360	SEMP1(h)	136	334
MO000145361	claudin1(h)	136	337
MO000109635	TGC-isoform1(h)	244	340
MO000138273	DcR3(h)	192	341
MO000114110	TRB3(h)	178	343
MO000019826	NAP-2(h)	249	344
MO000110890	SL-1(h)	290	345
MO000132610	SCCA1(h)	194	346
MO000216036	NCAM1-isoform1(h)	142	346
MO000216039	NCAM1-isoform3(h)	142	346
MO000216040	NCAM1-isoform4(h)	142	346
MO000216041	NCAM1-isoform5(h)	142	346
MO000216042	NCAM1-isoform6(h)	142	346
MO000214574	LY6D(h)	76	349
MO000216038	NCAM1-isoform2(h)	142	351
MO000032453	ANG-2(h)	158	352
MO000134787	ANG-2-isoform1(h)	158	352
MO000216037	NCAM1(h)	142	352
MO000254205	ANG-2-isoform2(h)	158	352
MO000254206	ANG-2-isoform3(h)	158	352
MO000018487	AT1A(h)	165	355
MO000210748	FKBP10(h)	186	358
MO000223910	TNFSF9(h)	349	358
MO000019817	CXCR1(h)	253	359
MO000019425	IL-11(h)	97	361
MO000199867	OTR(h)	96	362
MO000312209	IL-11-isoform2(h)	97	362
MO000210747	FKBP10-isoform1(h)	186	366
MO000334584	FKBP10-isoform2(h)	186	366
MO000028695	Twist-1(h)	156	368
MO000107906	RNF125(h)	69	370

MO000128200	claudin2(h)	173	373
MO000113967	IMP-1-isoform1(h)	310	376
MO000126463	alpha-fetoprotein(h)	21	376
MO000286402	IMP-1-isoform2(h)	310	376
MO000132077	BCMA(h)	263	377
MO000067827	IMP-1(h)	310	378
MO000132608	SCCA1-isoform1(h)	194	378
MO000257531	SCCA1-isoform2(h)	194	378
MO000063137	Grp(h)	266	379
MO000083592	E2F-1(h)	49	379
MO000292757	Cdk4:AMPKalpha-2:AMPKbeta-1{myr}:AMPKgamma-1:(AMP)3	77	379
MO000277984	AMPKalpha-2:AMPKbeta-1{myr}:AMPKgamma-1:(AMP)3	77	381
MO000277983	AMPKalpha-2:AMPKbeta-1{myr}:AMPKgamma-1:AMP:(ATP)2	77	382
MO000277982	AMPKalpha-2:AMPKbeta-1{myr}:AMPKgamma-1	77	383
MO000019834	GROalpha(h)	162	385
MO000025665	NF-E2 p45(h)	58	386
MO000061587	col1A1(h)	197	386
MO000097603	NF-E2 p45(h){sumoK368}	58	386
MO000020480	activin A(h)	150	387
MO000165657	activinA(h)	150	388
MO000480324	E-selectin(h){pY603}	138	393
MO000122552	E-selectin(h)	138	395
MO000256555	LOX-1-isoform2(h)	277	395
MO000286626	LOX-1-isoform3(h)	277	395
MO000180154	LOX-1(h)	277	397
MO000211835	Grp-isoform1(h)	266	397
MO000211836	Grp-isoform2(h)	266	397
MO000211837	Grp-isoform3(h)	266	397
MO000166857	IL-23p19(h):IL-12p40(h)	169	408
MO000068799	DEFA3(h)	281	410
MO000165356	IL-23p19(h)	169	410
MO000022339	IL-8(h):CXCR1(h):G-alpha-i2(h)	253	412
MO000255409	GHR-isoform2(h)	305	412
MO000255410	GHR-isoform3(h)	305	412
MO000255411	GHR-isoform4(h)	305	412
MO000041798	TGC(h)	244	413
MO000109636	TGC-isoform2(h)	244	413
MO000109637	TGC-isoform3(h)	244	413
MO000132075	BCMA-isoform1(h)	263	413
MO000319208	BCMA-isoform2(h)	263	413
MO000022280	IL-8(h):CXCR1(h)	253	415
MO000041266	IL-8:CXCR1{p}	253	415
MO000059826	GHR-isoform1(h)	305	415
MO000102880	ptges(h)	285	415
MO000166804	LDP4-isoform1(h)	270	415
MO000166808	LDP4-isoform2(h)	270	415
MO000170336	CXCR1(h):IL-8(h)	253	415
MO000005809	GHR(h)	305	416
MO000166805	LDP4(h)	270	417
MO000109699	tpx2-isoform1(h){ub}n	108	424
MO000117217	granzymeB(h)	117	425
MO000222105	SPRR2A(h)	172	428
MO000117020	AMPKgamma-1:AMPKbeta:AMPKalpha{pT172}	77	429
MO000125585	Osteopontin-isoform1(h)	342	429
MO000125587	Osteopontin(h)	342	429
MO000125588	Osteopontin-isoform2(h)	342	429
MO000125589	Osteopontin-isoform3(h)	342	429
MO000125590	Osteopontin-isoform4(h)	342	429
MO000173781	AMPKgamma-1:AMPKbeta:AMPKalpha{pT172}:mTOR:raptor:mLST8:PRAS40:ulk1:APG13:RB1CC1	77	429
MO000178073	AMPKalpha{pT172}:AMPKbeta:AMPKgamma-1:mTOR:raptor{pS722}{pS792}:mLST8:RB1CC1:PRAS40:ulk1:APG13	77	429
MO000257917	TRIM29-Beta(h)	93	429
MO000271420	Osteopontin-isoform5(h)	342	429
MO000079959	IL-2(h)	140	430
MO000124621	TRIM29-alpha(h)	93	430
MO000124622	TRIM29(h)	93	431
MO000032677	trkB(h)	374	432
MO000256495	trkB-T1(h)	374	432
MO000256496	trkB-T-Shc(h)	374	432
MO000256497	trkB-isoform4(h)	374	432
MO000256498	trkB-isoform5(h)	374	432
MO000256499	trkB-T-TK(h)	374	432
MO000256500	trkB-N-T1(h)	374	432
MO000333036	IL-13Ralpha2(h)	20	437
MO000044348	SNAI1(h)	163	438
MO000057790	cathepsin G(h)	403	439
MO000026137	MSX-1(h)	122	440
MO000122290	CD90(h)	431	445

MO000035572	akap5(h)	225	449
MO000097270	group 10 secretory phospholipase A2(h)	441	449
MO000224339	THBS2(h)	86	450
MO000114109	TRB3(h)	178	452
MO000321217	(MCP-1)2:CCR2:Gq:GDP	22	452
MO000123125	cyclosome{p}n:Cdc20:(MAD2)2:BubR1:cyclinB1:Cdk1:Cks1:p31-comet:(E2-C{ub(1)})n	416	453
MO000123384	PTTG1:Separin{pS1126}:cyclosome{p}n:Fzr1:E2-C{ub(1)}	416	454
MO000123226	Nek2A:cyclosome{p}n:Cdc20:E2-C{ub(1)}	416	455
MO000028384	NeuroD1(h)	198	456
MO000041426	MLK2:HTT:hap1:NeuroD	198	456
MO000056464	NeuroD1(h){ace}	198	456
MO000123290	cyclosome{p}n:Fzr1:cyclinB1:E2-C{ub(1)}	416	456
MO000123308	cyclosome{p}n:Fzr1:cyclinB1{ub(1)}:(E2-C{ub(1)})n	416	456
MO000022283	CXCR2(h)	364	457
MO000022291	CXCR2(h){p}	364	457
MO000123239	Nek2A{ub(1)}:cyclosome{p}n:Cdc20:(E2-C{ub(1)})n	416	458
MO000123386	PTTG1{ub(1)}:Separin{pS1126}:cyclosome{p}n:Fzr1:(E2-C{ub(1)})n	416	459
MO000043523	E2-C(h)	416	460
MO000104531	E2-C-isoform1(h)	416	460
MO000113663	E2-C(h){ub}n	416	460
MO000287020	E2-C-isoform2(h)	416	460
MO000287021	E2-C-isoform3(h)	416	460
MO000287022	E2-C-isoform4(h)	416	460
MO000069842	kallikrein-3(h)	51	461
MO000110915	Matrin(h)	405	461
MO000081810	kallikrein-3-isoform1(h)	51	462
MO000286466	kallikrein-3-isoform2(h)	51	462
MO000286467	kallikrein-3-isoform3(h)	51	462
MO000286468	kallikrein-3-isoform4(h)	51	462
MO000319007	kallikrein-3-isoform5(h)	51	462
MO000129907	Frzb-1(h)	189	466
MO000197388	TSA1(h)	38	467
MO000038398	Raf-1:MEK2:ERK:KSR	232	468
MO000042726	Ajuba(h)	183	469
MO000082884	Ajuba-isoform1(h)	183	469
MO000277174	Ajuba-isoform2(h)	183	469
MO000139291	PAP1(h)	68	484
MO000128346	kallikrein-6-isoform1(h)	314	487
MO000032617	NR2B(h)	157	491
MO000043123	GLP-1(h)	229	491
MO000032604	glucagon(h)	229	492
MO000035182	MRP-14(h)	350	498
MO000138972	GCG(h)	229	498
MO000114796	AMPKalpha(h){pT}	77	499
MO000023447	Cdc25B(h)	432	502
MO000030907	Cdc25B(h):p38alpha(h)	432	502
MO000030908	Cdc25B(h){p}	432	502
MO000030940	Cdc25B(h):Chk1(h)	432	502
MO000030995	Raf-1(h):Cdc25B(h)	432	502
MO000031033	Cdc25B-isoform1(h)	432	502
MO000031034	Cdc25B-isoform2(h)	432	502
MO000031035	Cdc25B-isoform3(h)	432	502
MO000114162	CB1-isoform1(h)	381	502
MO000254730	CB1-isoform2(h)	381	502
MO000254731	CB1-isoform3(h)	381	502
MO000256202	Cdc25B-isoform4(h)	432	502
MO000036098	TIMP-1(h)	242	503
MO000018038	CB1(h)	381	505
MO000039028	activin A:(ActR-II:ActR-IB{pS}{pT})2	150	509
MO000039029	activin A:(ActR-II:ActR-IB)2	150	510
MO000039030	activin A:(ActR-II)2	150	511
MO000056875	CXCR1(h)	253	520
MO000119842	LIN7A(h)	149	521
MO000022340	IL-8(h):CXCR2(h):G-alpha-i2(h)	364	522
MO000022284	IL-8(h):CXCR2(h)	364	524
MO000001760	Blk(h)	206	525
MO000041241	(CXCR2(h))2	364	525
MO000098824	xanthine dehydrogenase/oxidase(h)	300	525
MO000171737	saa1(h)	315	526
MO000021365	SL-1(h)	290	530
MO000225595	ZBTB7C(h)	193	533
MO000217740	PPY(h)	175	541
MO000034900	Nedd4-2(h)	331	545
MO000058631	Nedd4-2-xbb1(h)	331	545
MO000058639	Nedd4-2-isoform4(h)	331	545
MO000058647	Nedd4-2-isoform5(h)	331	545
MO000058653	Nedd4-2-isoform1(h)	331	545

MO000058658	Nedd4-2-isoform6(h)	331	545
MO000058660	Nedd4-2-isoform7(h)	331	545
MO000256326	Nedd4-2-isoform3(h)	331	545
MO000271336	Nedd4-2-isoform9(h)	331	545
MO000021670	T3R-beta1(h)	207	546
MO000038356	Fz:Wnt	273	552
MO000023258	wif1(h)	275	553
MO000133252	SNAI1(h)	163	553
MO000208489	CST2(h)	190	557
MO000142626	I-TAC(h)	120	559
MO000217739	PPY-isoform1(h)	175	560
MO000340846	PPY-isoform2(h)	175	560
MO000096389	COX2(h)	283	568
MO000038143	Fibrinogen(h)	356	569
MO000328367	Beta-tubulin3-isoform2(h)	250	570
MO000127277	Beta-tubulin3(h)	250	571
MO000138445	EAB1-isoform1(h)	259	571
MO000138447	EAB1(h)	259	571
MO000255203	EAB1-isoform2(h)	259	571
MO000255204	EAB1-isoform3(h)	259	571
MO000214070	LGI1(h)	407	575
MO000137676	CR-1(h)	328	582
MO000161143	Beta-klotho(h)	313	583
MO000214069	LGI1-isoform1(h)	407	593
MO000214071	LGI1-isoform2(h)	407	593
MO000214072	LGI1-isoform3(h)	407	593
MO000094840	tryptophan 5-hydroxylase 1-isoform1(h)	226	594
MO000094841	tryptophan 5-hydroxylase 1(h)	226	594
MO000094845	tryptophan 5-hydroxylase 1-isoform2(h)	226	594
MO000166835	cacna1a(h)	234	599
MO000137675	CR-1(h)	328	600
MO000189898	CLCA1(h)	243	601
MO000033776	PGC-1-isoform1(h)	214	607
MO000057837	PGC-1(h)	214	607
MO000319127	PGC-1-isoform2(h)	214	607
MO000319266	PGC-1(h){aceK}	214	607
MO000328143	PGC-1-B5(h)	214	607
MO000328144	PGC-1-B4(h)	214	607
MO000328145	PGC-1-B4-8a(h)	214	607
MO000328146	PGC-1-B5-NT(h)	214	607
MO000328147	PGC-1-B4-3ext(h)	214	607
MO000328148	PGC-1-8a(h)	214	607
MO000328149	PGC-1-isoform9(h)	214	607
MO000110916	Matrin(h)	405	608
MO000083262	TSLP-isoform1(h)	180	611
MO000340258	TSLP-isoform2(h)	180	611
MO000083263	TSLP(h)	180	613
MO000166837	cacna1a-isoform3(h)	234	613
MO000166838	cacna1a-isoform4(h)	234	613
MO000166839	cacna1a-isoform5(h)	234	613
MO000166840	cacna1a-isoform6(h)	234	613
MO000286087	cacna1a-isoform1(h)	234	613
MO000035183	MRP-8(h)	241	614
MO000166836	cacna1a-isoform2(h)	234	618
MO000102280	p64Shc3(h)	317	626
MO000028382	ATH-1(h)	385	627
MO000102282	p52Shc3(h)	317	627
MO000094445	amine oxidase (flavin-containing) A(h)	352	628
MO000102281	Shc-3(h)	317	628
MO000189677	RENOX-isoform1(h)	386	629
MO000189679	RENOX-isoform2(h)	386	629
MO000189680	RENOX-isoform3(h)	386	629
MO000189681	RENOX-isoform4(h)	386	629
MO000189682	RENOX-isoform5(h)	386	629
MO000189684	RENOX-isoform7(h)	386	629
MO000328004	RENOX-isoform8(h)	386	629
MO000328005	RENOX-isoform9(h)	386	629
MO000213605	KLK15-isoform1(h)	208	631
MO000213607	KLK15-isoform2(h)	208	631
MO000213608	KLK15-isoform3(h)	208	631
MO000213609	KLK15-isoform4(h)	208	631
MO000327871	KLK15-isoform5(h)	208	631
MO000112603	F-Spondin(h)	351	634
MO000056873	CXCR2(h)	364	635
MO000213606	KLK15(h)	208	636
MO000189683	RENOX-isoform6(h)	386	637
MO000021387	FGF-19(h)	370	638

MO000189678	RENOX(h)	386	638
MO000132309	CRTL1(h)	252	639
MO000208973	DKK2(h)	276	639
MO000286013	amine oxidase (flavin-containing) A-isoform2(h)	352	641
MO000096009	creatine kinase B-type(h)	286	643
MO000203518	SCIN(h)	301	649
MO000203517	SCIN-isoform1(h)	301	650
MO000203519	SCIN-isoform2(h)	301	650
MO000203520	SCIN-isoform3(h)	301	650
MO000212214	HHIP-isoform1(h)	287	661
MO000212216	HHIP-isoform2(h)	287	661
MO000270794	nAChR-alpha7-isoform2(h)	320	661
MO000277172	nAChR-alpha7-isoform3(h)	320	661
MO000038742	Nedd4-2:ENaC	331	662
MO000038743	SGK-1{pT256}:Nedd4-2:ENaC	331	663
MO000134463	nAChR-alpha7(h)	320	663
MO000212215	HHIP(h)	287	663
MO000032760	RGS13(h)	248	666
MO000129939	PRL3-isoform3(h)	323	667
MO000129932	PRL3-isoform1(h)	323	668
MO000257869	PRL3-isoform2(h)	323	669
MO000129933	PRL3(h)	323	670
MO000064565	PLA2G2A(h)	282	674
MO000166313	wnt5a-isoform1(h)	396	675
MO000166314	wnt5a(h)	396	675
MO000166315	wnt5a-isoform2(h)	396	675
MO000100687	SIM2s(h)	316	687
MO000018073	Kir2.2(h)	355	688
MO000156225	PRDC(h)	327	689
MO000038146	fibrinogen-beta(h)	356	691
MO000021449	MR(h)	411	695
MO000187836	wnt3(h)	273	707
MO000041581	FRP(h)	330	708
MO000103482	MR-isoform3(h)	411	708
MO000103485	MR-isoform4(h)	411	708
MO000018623	NPY1-R(h)	402	709
MO000082290	MR-isoform1(h)	411	710
MO000103481	MR-isoform2(h)	411	710
MO000018438	PAR3(h)	297	737
MO000151438	PAR3-isoform1(h)	297	737
MO000286648	PAR3-isoform2(h)	297	737
MO000255647	IL1R2-Short(h)	348	739
MO000038846	Fibrinogen:(alpha IIb beta3)2:(Src{pY419})2:(CIB)2:(Ca)2:(talin)2:(Syk{pY})2	356	760
MO000129279	apolipoprotein-H(h)	404	760
MO000038845	Fibrinogen:(alpha IIb beta3)2:(Src{pY419})2:(CIB)2:(Ca)2:(talin)2:(Syk)2	356	761
MO000038842	Fibrinogen:(alpha IIb beta3)2:(Src{pY419})2:(CIB)2:(Ca)2:(talin)2	356	762
MO000038841	Fibrinogen:(alpha IIb beta3)2:(Src)2:(CIB)2:(Ca)2:(talin)2	356	763
MO000038839	Fibrinogen:(alpha IIb beta3)2:(Src)2	356	764
MO000038838	Fibrinogen:(alpha IIb beta3)2:(Src{pY529})2	356	765
MO000166304	wnt2(h)	354	789
MO000125052	cGKII-isoform1(h)	371	807
MO000334730	cGKII-isoform2(h)	371	807
MO000125053	cGKII(h)	371	809
MO000143095	ESM-1-isoform1(h)	399	818
MO000271046	ESM-1-isoform2(h)	399	818
MO000032623	PC5(h)	442	819
MO000143096	ESM-1(h)	399	820
MO000078608	PC5B(h)	442	830
MO000256696	PC5A(h)	442	830
MO000293886	EloC:EloB:Cul-2:Roc1:E2	416	859
MO000293887	HIF-1alpha:(RACK1{pS146})2:SSAT:EloC:EloB:Cul-2:Roc1:E2	416	859

Supplementary Table 5A. Master molecules found in RSS1 and their ranks.

RSS2 Master Molecules			
ID	Master molecule name	CMA rank	Total rank
MO000082741	IL-1alpha(h)	3	17
MO000019374	IL-1alpha(h)	3	22
MO000038335	fMLP-R:Gi:GDP	12	28
MO000038336	fMLP:fMLP-R:Gi:GDP	12	39
MO000125815	FCgammaRIIIA(h)	68	83
MO000023462	COX2(h)	70	89
MO000110915	Matrin(h)	61	106
MO000036091	G-CSF(h)	83	111
MO000130281	G-CSF-isoform1(h)	83	112
MO000286201	G-CSF-isoform3(h)	83	112
MO000151288	G-CSF-isoform2(h)	83	114
MO000125816	FCgammaRIIIA(h)	68	115
MO000166804	LDP4-isoform1(h)	87	137
MO000166808	LDP4-isoform2(h)	87	137
MO000166805	LDP4(h)	87	141
MO000180153	LOX-1-isoform1(h)	136	154
MO000022283	CXCR2(h)	92	159
MO000022291	CXCR2(h){p}	92	159
MO000110890	SL-1(h)	123	167
MO000214069	LGI1-isoform1(h)	52	186
MO000214071	LGI1-isoform2(h)	52	186
MO000214072	LGI1-isoform3(h)	52	186
MO000207255	CHGA(h)	66	187
MO000214070	LGI1(h)	52	189
MO000033461	fMLP-R(h)	176	202
MO000145360	SEMP1(h)	28	202
MO000114109	TRB3(h)	22	204
MO000145361	claudin1(h)	28	205
MO000007287	inhibin beta A(h)	218	219
MO000036116	GM-CSF(h)	202	219
MO000070193	OSR1(h)	11	229
MO000132077	BCMA(h)	179	240
MO000110916	Matrin(h)	61	241
MO000019817	CXCR1(h)	141	242
MO000114110	TRB3(h)	22	244
MO000132075	BCMA-isoform1(h)	179	244
MO000319208	BCMA-isoform2(h)	179	244
MO000036098	TIMP-1(h)	24	245
MO000000322	G12/13	12	246
MO000256555	LOX-1-isoform2(h)	136	249
MO000286626	LOX-1-isoform3(h)	136	249
MO000180154	LOX-1(h)	136	251
MO000171737	saa1(h)	119	252
MO000035415	IL-2(h)	235	254
MO000125306	MDA7-isoform1(h)	249	254
MO000271183	MDA7-isoform2(h)	249	254
MO000271184	MDA7-isoform3(h)	249	254
MO000271185	MDA7-isoform4(h)	249	254
MO000032617	NR2B(h)	25	255
MO000097270	group 10 secretory phospholipase A2(h)	243	255
MO000022340	IL-8(h):CXCR2(h):G-alpha-i2(h)	92	256
MO000022284	IL-8(h):CXCR2(h)	92	258
MO000041241	(CXCR2(h))2	92	259
MO000125307	MDA7(h)	249	259
MO000018285	Gq:GDP	12	260
MO000322608	(angiotensin II)2:(AT1A)2:Gq:GDP	12	264
MO000114255	AMPKalpha-2(h)	103	265
MO000222105	SPRR2A(h)	2	268
MO000255409	GHR-isoform2(h)	170	278
MO000255410	GHR-isoform3(h)	170	278
MO000255411	GHR-isoform4(h)	170	278
MO000059826	GHR-isoform1(h)	170	281
MO000005809	GHR(h)	170	282
MO000113967	IMP-1-isoform1(h)	184	288
MO000286402	IMP-1-isoform2(h)	184	288
MO000056873	CXCR2(h)	92	289
MO000067827	IMP-1(h)	184	291
MO000128200	claudin2(h)	120	296
MO000199867	OTR(h)	34	299
MO000019852	I-TAC(h)	207	302
MO000019977	MIP-3beta(h)	201	303
MO000021783	mmp2(h)	125	303
MO000138687	GP39(h)	121	304
MO000122290	CD90(h)	286	305
MO000022339	IL-8(h):CXCR1(h):G-alpha-i2(h)	141	306

MO000031992	granzymeB(h)	204	307
MO000022280	IL-8(h):CXCR1(h)	141	309
MO000041266	IL-8:CXCR1{p}	141	309
MO000122634	drebrin(h)	26	309
MO000170336	CXCR1(h):IL-8(h)	141	309
MO000089174	NF-E2 p45(h)	6	322
MO000334232	M1-isoform2(h)	278	330
MO000060023	M1-isoform1(h)	278	331
MO000221557	IBSP(h)	157	332
MO000018765	M1(h)	278	333
MO000056875	CXCR1(h)	141	334
MO000025665	NF-E2 p45(h)	6	335
MO000097603	NF-E2 p45(h){sumoK368}	6	335
MO000028384	NeuroD1(h)	98	338
MO000041426	MLK2:HTT:hap1:NeuroD	98	338
MO000056464	NeuroD1(h){ace}	98	338
MO000223910	TNFSF9(h)	325	338
MO000021365	SL-1(h)	123	342
MO000033776	PGC-1-isoform1(h)	72	342
MO000057837	PGC-1(h)	72	342
MO000319127	PGC-1-isoform2(h)	72	342
MO000319266	PGC-1(h){aceK}	72	342
MO000328143	PGC-1-B5(h)	72	342
MO000328144	PGC-1-B4(h)	72	342
MO000328145	PGC-1-B4-8a(h)	72	342
MO000328146	PGC-1-B5-NT(h)	72	342
MO000328147	PGC-1-B4-3ext(h)	72	342
MO000328148	PGC-1-8a(h)	72	342
MO000328149	PGC-1-isoform9(h)	72	342
MO000128346	kallikrein-6-isoform1(h)	206	344
MO000038356	Fz:Wnt	35	345
MO000166313	wnt5a-isoform1(h)	35	345
MO000166314	wnt5a(h)	35	345
MO000166315	wnt5a-isoform2(h)	35	345
MO000041798	TGC(h)	197	352
MO000109636	TGC-isoform2(h)	197	352
MO000109637	TGC-isoform3(h)	197	352
MO000096389	COX2(h)	70	354
MO000138273	DcR3(h)	234	354
MO000109635	TGC-isoform1(h)	197	355
MO000017291	integrins	10	359
MO000126463	alpha-fetoprotein(h)	9	360
MO000061587	col1A1(h)	225	365
MO000080123	GM-CSF(h)	202	365
MO000059884	CD22(h){pY}	148	371
MO000480316	CD22(h){pY777}	148	371
MO000057790	cathepsin G(h)	324	372
MO000059880	CD22(h)	148	373
MO000206060	CD22-alpha(h)	148	373
MO000286128	CD22-isoform3(h)	148	373
MO000311960	CD22-isoform4(h)	148	373
MO000327621	CD22-isoform5(h)	148	373
MO000480324	E-selectin(h){pY603}	182	381
MO000122552	E-selectin(h)	182	382
MO000286013	amine oxidase (flavin-containing) A-isoform2(h)	211	382
MO000094445	amine oxidase (flavin-containing) A(h)	211	383
MO000210748	FKBP10(h)	256	395
MO000292757	Cdk4:AMPKalpha-2:AMPKbeta-1{myr}:AMPKgamma-1:(AMP)3	103	398
MO000292758	(angiotensin II)2:(AT2 receptor)2:Cdk4:AMPKalpha-2:AMPKbeta-1{myr}:AMPKgamma-1:(AMP)3	103	398
MO000277984	AMPKalpha-2:AMPKbeta-1{myr}:AMPKgamma-1:(AMP)3	103	400
MO000277983	AMPKalpha-2:AMPKbeta-1{myr}:AMPKgamma-1:AMP:(ATP)2	103	401
MO000020480	activin A(h)	218	402
MO000220460	S100A12(h)	391	402
MO000277982	AMPKalpha-2:AMPKbeta-1{myr}:AMPKgamma-1	103	402
MO000165657	activinA(h)	218	403
MO000210747	FKBP10-isoform1(h)	256	408
MO000334584	FKBP10-isoform2(h)	256	408
MO000021785	gelatinaseB(h)	30	410
MO000166857	IL-23p19(h):IL-12p40(h)	166	411
MO000138322	ET3(h)	27	412
MO000167179	ptprh(h)	64	412
MO000032592	BDNF(h)	374	413
MO000165356	IL-23p19(h)	166	413
MO000254403	BDNF-isoform3(h)	374	414
MO000254404	BDNF-isoform4(h)	374	414
MO000254405	BDNF-isoform5(h)	374	414
MO000043202	TFF1(h)	133	415

MO000125585	Osteopontin-isoform1(h)	288	415
MO000125587	Osteopontin(h)	288	415
MO000125588	Osteopontin-isoform2(h)	288	415
MO000125589	Osteopontin-isoform3(h)	288	415
MO000125590	Osteopontin-isoform4(h)	288	415
MO000211835	Grp-isoform1(h)	299	415
MO000211836	Grp-isoform2(h)	299	415
MO000211837	Grp-isoform3(h)	299	415
MO000271420	Osteopontin-isoform5(h)	288	415
MO000019424	IL-6(h)	398	417
MO000254402	BDNF-isoform2(h)	374	417
MO000063137	Grp(h)	299	418
MO000103495	BDNF-isoform1(h)	374	420
MO000161143	Beta-klotho(h)	224	420
MO000123125	cyclosome{p}n:Cdc20:(MAD2)2:BubR1:cyclinB1:Cdk1:Cks1:p31-comet:(E2-C{ub(1)})n	352	422
MO000044348	SNAI1(h)	155	423
MO000123384	PTTG1:Separin{pS1126}:cyclosome{p}n:Fzr1:E2-C{ub(1)}	352	423
MO000123226	Nek2A:cyclosome{p}n:Cdc20:E2-C{ub(1)}	352	424
MO000129939	PRL3-isoform3(h)	57	424
MO000089119	IL-11-isoform1(h)	401	425
MO000123290	cyclosome{p}n:Fzr1:cyclinB1:E2-C{ub(1)}	352	425
MO000123308	cyclosome{p}n:Fzr1:cyclinB1{ub(1)}:(E2-C{ub(1)})n	352	425
MO000129932	PRL3-isoform1(h)	57	425
MO000021305	TGFbeta2(h)	43	426
MO000257869	PRL3-isoform2(h)	57	426
MO000123239	Nek2A{ub(1)}:cyclosome{p}n:Cdc20:(E2-C{ub(1)})n	352	427
MO000129933	PRL3(h)	57	427
MO000123386	PTTG1{ub(1)}:Separin{pS1126}:cyclosome{p}n:Fzr1:(E2-C{ub(1)})n	352	428
MO000043523	E2-C(h)	352	429
MO000104531	E2-C-isoform1(h)	352	429
MO000113663	E2-C(h){ub}n	352	429
MO000287020	E2-C-isoform2(h)	352	429
MO000287021	E2-C-isoform3(h)	352	429
MO000287022	E2-C-isoform4(h)	352	429
MO000213605	KLK15-isoform1(h)	46	432
MO000213607	KLK15-isoform2(h)	46	432
MO000213608	KLK15-isoform3(h)	46	432
MO000213609	KLK15-isoform4(h)	46	432
MO000327871	KLK15-isoform5(h)	46	432
MO000096672	OSM(h)	190	433
MO000068799	DEFA3(h)	312	435
MO000098824	xanthine dehydrogenase/oxidase(h)	245	435
MO000167178	ptprh-isoform1(h)	64	436
MO000321217	(MCP-1)2:CCR2:Gq:GDP	12	437
MO000213606	KLK15(h)	46	438
MO000121062	IGFBP-5(h)	67	442
MO000135001	calcitonin-isoform2(h)	443	445
MO000205368	calcitonin-isoform3(h)	443	445
MO000205372	calcitonin-isoform1(h)	443	445
MO000030861	MKP-5(h)	240	446
MO000126134	MKP-5-isoform1(h)	240	447
MO000143095	ESM-1-isoform1(h)	39	447
MO000271046	ESM-1-isoform2(h)	39	447
MO000189898	CLCA1(h)	102	448
MO000114162	CB1-isoform1(h)	393	449
MO000143096	ESM-1(h)	39	449
MO000254730	CB1-isoform2(h)	393	449
MO000254731	CB1-isoform3(h)	393	449
MO000018623	NPY1-R(h)	187	451
MO000018038	CB1(h)	393	452
MO000032453	ANG-2(h)	266	452
MO000032593	calcitonin(h)	443	452
MO000134787	ANG-2-isoform1(h)	266	452
MO000254205	ANG-2-isoform2(h)	266	452
MO000254206	ANG-2-isoform3(h)	266	452
MO000079959	IL-2(h)	235	455
MO000038742	Nedd4-2:ENaC	131	458
MO000038743	SGK-1{pT256}:Nedd4-2:ENaC	131	459
MO000083263	TSLP(h)	31	461
MO000021670	T3R-beta1(h)	147	462
MO000043123	GLP-1(h)	271	462
MO000032604	glucagon(h)	271	463
MO000117020	AMPKgamma-1:AMPKbeta:AMPKalpha{pT172}	103	464
MO000117518	MT3-MMP(h)	231	464
MO000173781	AMPKgamma-1:AMPKbeta:AMPKalpha{pT172}:mTOR:raptor:mLST8:PRAS40:ulk1:APG13:RB1CC1	103	464
MO000178073	AMPKalpha{pT172}:AMPKbeta:AMPKgamma-1:mTOR:raptor{pS722}{pS792}:mLST8:RB1CC1:PRAS40:ulk1:APG13	103	464
MO000129279	apolipoprotein-H(h)	101	465

MO000138972	GCG(h)	271	466
MO000032760	RGS13(h)	56	469
MO000035183	MRP-8(h)	90	472
MO000046121	Aurora-A{pT288}:tpx2{pS}	382	474
MO000046129	PP1:Aurora-A{pT288}:tpx2{pS}	382	474
MO000064565	PLA2G2A(h)	63	475
MO000042660	tpx2(h){pS}	382	476
MO000159782	LEF-1(h)	140	477
MO000042658	tpx2(h):Aurora-A(h)	382	478
MO000042656	tpx2(h)	382	479
MO000046123	importin-alpha:importin-beta:tpx2:Kid	382	479
MO000109695	tpx2-isoform1(h)	382	479
MO000281079	mmp2-isoform2(h)	125	479
MO000286538	mmp2-isoform3(h)	125	479
MO000339227	tpx2-isoform2(h)	382	479
MO000032677	trkB(h)	448	480
MO000256495	trkB-T1(h)	448	480
MO000256496	trkB-T-Shc(h)	448	480
MO000256497	trkB-isoform4(h)	448	480
MO000256498	trkB-isoform5(h)	448	480
MO000256499	trkB-T-TK(h)	448	480
MO000256500	trkB-N-T1(h)	448	480
MO000117753	mmp2-isoform1(h)	125	481
MO000083262	TSLP-isoform1(h)	31	482
MO000340258	TSLP-isoform2(h)	31	482
MO000007322	IL-13(h)	459	484
MO000004594	EphB1(h)	426	486
MO000137371	EphB1-isoform1(h)	426	488
MO000334533	EphB1-isoform2(h)	426	488
MO000334534	EphB1-isoform3(h)	426	488
MO000102880	ptges(h)	338	492
MO000215287	MMP12(h)	313	492
MO000042726	Ajuba(h)	208	493
MO000082884	Ajuba-isoform1(h)	208	493
MO000277174	Ajuba-isoform2(h)	208	493
MO000035182	MRP-14(h)	335	494
MO000018675	IP(h)	5	497
MO000110307	ETV4-isoform1(h)	18	503
MO000286299	ETV4-isoform2(h)	18	503
MO000334550	ETV4-isoform3(h)	18	503
MO000065940	CD11C(h)	42	504
MO000138503	CD20(h)	1	504
MO000046009	ETV4(h)	18	506
MO000102280	p64Shc3(h)	215	506
MO000102282	p52Shc3(h)	215	507
MO000102281	Shc-3(h)	215	508
MO000018534	GAL1(h)	91	515
MO000125976	CD21-isoform-A(h)	314	515
MO000125982	CD21-isoform-C(h)	314	515
MO000125983	CD21-isoform-D(h)	314	515
MO000117217	granzymeB(h)	204	518
MO000125979	CD21-isoform-B(h)	314	518
MO000125978	CD21(h)	314	519
MO000114796	AMPKalpha(h){pT}	103	520
MO000137675	CR-1(h)	328	526
MO000208973	DKK2(h)	188	526
MO000117396	MT1-MMP(h)	297	529
MO000328367	Beta-tubulin3-isoform2(h)	213	530
MO000061333	CD89(h)	130	531
MO000127277	Beta-tubulin3(h)	213	531
MO000083701	TGFbeta-2A(h)	43	532
MO000083706	TGFbeta-2B(h)	43	533
MO000019375	IL-1beta(h)	303	534
MO000019826	NAP-2(h)	465	534
MO000209228	DUSP14(h)	244	534
MO000021420	angiotensin II(h)	486	535
MO000133252	SNAI1(h)	155	536
MO000187923	AMPKalpha-1{pT183}:AMPKalpha-2:sestrin2	103	536
MO000187920	sestrin2:AMPKalpha-1:AMPKalpha-2	103	537
MO000021449	MR(h)	330	538
MO000080033	IL-6(h)	398	539
MO000112603	F-Spondin(h)	276	543
MO000141169	lithostathine1alpha(h)	424	546
MO000028382	ATH-1(h)	265	554
MO000117516	MT3-MMP-L(h)	231	554
MO000117517	MT3-MMP-S(h)	231	554
MO000255021	MKP-5-isoform2(h)	240	560

MO000019425	IL-11(h)	401	561
MO000019903	MIP-1delta(h)	178	562
MO000203518	SCIN(h)	226	562
MO000312209	IL-11-isoform2(h)	401	562
MO000019426	OSM(h)	190	564
MO000016612	TLR2(h)	322	566
MO000103482	MR-isoform3(h)	330	566
MO000103485	MR-isoform4(h)	330	566
MO000078608	PC5B(h)	192	568
MO000103481	MR-isoform2(h)	330	568
MO000203517	SCIN-isoform1(h)	226	568
MO000203519	SCIN-isoform2(h)	226	568
MO000203520	SCIN-isoform3(h)	226	568
MO000255647	IL1R2-Short(h)	168	568
MO000256696	PC5A(h)	192	568
MO000082290	MR-isoform1(h)	330	569
MO000225595	ZBTB7C(h)	230	569
MO000032623	PC5(h)	192	570
MO000025969	WT1 -KTS(h)	430	572
MO000025970	WT1 I(h)	430	572
MO000025971	WT1-del2(h)	430	572
MO000025972	WT1 I-del2(h)	430	572
MO000156225	PRDC(h)	237	572
MO000258120	WT1-isoform6(h)	430	572
MO000258121	WT1-isoform7(h)	430	572
MO000258122	WT1-isoform8(h)	430	572
MO000271719	WT1-isoform9(h)	430	572
MO000039028	activin A:(ActR-II:ActR-IB{pS}{pT})2	218	575
MO000019762	FGF-1(h)	129	576
MO000039029	activin A:(ActR-II:ActR-IB)2	218	576
MO000028695	Twist-1(h)	277	577
MO000039030	activin A:(ActR-II)2	218	577
MO000025253	WT1 I -KTS(h)	430	580
MO000102040	WT1(h)	430	581
MO000021021	VRP(h)	175	586
MO000023447	Cdc25B(h)	503	586
MO000030907	Cdc25B(h):p38alpha(h)	503	586
MO000030908	Cdc25B(h){p}	503	586
MO000030940	Cdc25B(h):Chk1(h)	503	586
MO000030995	Raf-1(h):Cdc25B(h)	503	586
MO000031033	Cdc25B-isoform1(h)	503	586
MO000031034	Cdc25B-isoform2(h)	503	586
MO000031035	Cdc25B-isoform3(h)	503	586
MO000256202	Cdc25B-isoform4(h)	503	586
MO000217740	PPY(h)	259	590
MO000198687	periostin-isoform1(h)	158	596
MO000198689	periostin-isoform2(h)	158	596
MO000198690	periostin-isoform3(h)	158	596
MO000198691	periostin-isoform4(h)	158	596
MO000334975	periostin-isoform5(h)	158	596
MO000334976	periostin-isoform6(h)	158	596
MO000334977	periostin-isoform7(h)	158	596
MO000137676	CR-1(h)	328	597
MO000217739	PPY-isoform1(h)	259	599
MO000340846	PPY-isoform2(h)	259	599
MO000004685	ERK4(h)	495	601
MO000166304	wnt2(h)	169	601
MO000277891	AMPKalpha-2{pT172}:AMPKbeta-2{myr}:AMPKgamma-1:(AMP)3	103	601
MO000277890	AMPKalpha-2:AMPKbeta-2{myr}:AMPKgamma-1:(AMP)3	103	602
MO000198688	periostin(h)	158	603
MO000277889	AMPKalpha-2:AMPKbeta-2{myr}:AMPKgamma-1:AMP:(ATP)2	103	603
MO000277888	AMPKalpha-2:AMPKbeta-2{myr}:AMPKgamma-1	103	604
MO000281381	(angiotensin II)2:(AT2 receptor)2:(ATIP-isoform3)2:SHP-1	486	610
MO000281378	(angiotensin II)2:(AT2 receptor)2:(ATIP-isoform3)2	486	611
MO000280542	(angiotensin II)2:(AT2 receptor)2	486	612
MO000179101	cxcr3(h):IP-10(h)	195	617
MO000208489	CST2(h)	239	618
MO000003223	MT1-MMP(h)	297	619
MO000162954	galectin-2(h)	122	619
MO000094840	tryptophan 5-hydroxylase 1-isoform1(h)	294	626
MO000094841	tryptophan 5-hydroxylase 1(h)	294	626
MO000094845	tryptophan 5-hydroxylase 1-isoform2(h)	294	626
MO000059046	TFF1(h)	133	627
MO000026137	MSX-1(h)	309	628
MO000035572	akap5(h)	456	629
MO000001760	Blk(h)	336	630
MO000131941	ACLP(h)	306	631

MO000199871	VPAC1(h)	135	631
MO000145412	UBP41-isoform1(h)	281	633
MO000145413	UBP41(h)	281	634
MO000023258	wif1(h)	326	635
MO000197388	TSA1(h)	238	641
MO000139291	PAP1(h)	189	642
MO000320733	KLF4(h){ub}n	344	645
MO000255857	KLF4-isoform3(h)	344	647
MO000319000	KLF4-isoform4(h)	344	647
MO000319001	KLF4-isoform5(h)	344	647
MO000125555	KLF4-isoform1(h)	344	650
MO000125563	KLF4-isoform2(h)	344	651
MO000125561	KLF4(h)	344	652
MO000018523	ETA(h)	196	657
MO000097772	apoa2(h)	177	661
MO000125052	cGKII-isoform1(h)	246	664
MO000125053	cGKII(h)	246	664
MO000334730	cGKII-isoform2(h)	246	664
MO000010971	PDGFRbeta(h)	319	666
MO000019450	IL-1beta(h)	303	674
MO000034900	Nedd4-2(h)	466	675
MO000058631	Nedd4-2-xbb1(h)	466	675
MO000058639	Nedd4-2-isoform4(h)	466	675
MO000058647	Nedd4-2-isoform5(h)	466	675
MO000058653	Nedd4-2-isoform1(h)	466	675
MO000058658	Nedd4-2-isoform6(h)	466	675
MO000058660	Nedd4-2-isoform7(h)	466	675
MO000256326	Nedd4-2-isoform3(h)	466	675
MO000271336	Nedd4-2-isoform9(h)	466	675
MO000038398	Raf-1:MEK2:ERK:KSR	495	676
MO000059935	CD19(h)	327	677
MO000324193	PDGFRbeta-isoform1(h){pY686}{pY934}{pY970}	319	679
MO000109699	tpx2-isoform1(h){ub}n	382	683
MO000187836	wnt3(h)	254	685
MO000021387	FGF-19(h)	497	691
MO000019834	GROalpha(h)	461	696
MO000123641	NPY(h)	274	697
MO000059005	ERG(h)	255	701
MO000039099	IL-1beta-p17:IL-1RI:IL-1RAcP:MyD88:tollip:IRAK-1{pS376}{pT387}:IRAK-4:IRAK-2	303	708
MO000117465	FcgammaRIIA-isoform1(h)	462	708
MO000224339	THBS2(h)	337	710
MO000318859	CCL28-isoform2(h)	262	710
MO000135030	CCL28-isoform1(h)	262	711
MO000020080	CCL28(h)	262	712
MO000100687	SIM2s(h)	321	712
MO000132309	CRTL1(h)	310	712
MO000257989	UBP41-isoform3(h)	281	716
MO000257990	UBP41-isoform4(h)	281	716
MO000167596	UBP41-isoform2(h)	281	718
MO000034981	fibronectin(h)	405	726
MO000189677	RENOX-isoform1(h)	475	729
MO000189679	RENOX-isoform2(h)	475	729
MO000189680	RENOX-isoform3(h)	475	729
MO000189681	RENOX-isoform4(h)	475	729
MO000189682	RENOX-isoform5(h)	475	729
MO000189684	RENOX-isoform7(h)	475	729
MO000325893	MSX-2(h){ub}	275	729
MO000328004	RENOX-isoform8(h)	475	729
MO000328005	RENOX-isoform9(h)	475	729
MO000020862	VEGFR-2(h)	447	735
MO000281767	(angiotensin II)2:(AT1A{p})2:beta-arrestin2:Raf-1:MEK1:ERK2	486	735
MO000281768	(angiotensin II)2:(AT1A{p})2:beta-arrestin2:Raf-1:MEK1{pS218}{pS222}:ERK2{pT185}{pY187}	486	735
MO000189683	RENOX-isoform6(h)	475	737
MO000281766	(angiotensin II)2:(AT1A{p})2	486	737
MO000189678	RENOX(h)	475	738
MO000280543	(angiotensin II)2:(AT1A)2	486	738
MO000107906	RNF125(h)	460	741
MO000069842	kallikrein-3(h)	367	761
MO000081810	kallikrein-3-isoform1(h)	367	762
MO000286466	kallikrein-3-isoform2(h)	367	762
MO000286467	kallikrein-3-isoform3(h)	367	762
MO000286468	kallikrein-3-isoform4(h)	367	762
MO000319007	kallikrein-3-isoform5(h)	367	762
MO000032699	SOCS-3(h)	404	769
MO000099498	galactosylceramide sulfotransferase(h)	381	774
MO000129931	MMP-13(h)	270	774
MO000153812	BTLA1(h)	287	778

MO000087762	TLR2(h)	322	780
MO000333036	IL-13Ralpha2(h)	373	789
MO000119842	LIN7A(h)	425	791
MO000019888	MIP1A(h)	311	793
MO000021116	TGFbeta3(h)	380	806
MO000023404	FGF-BP(h)	392	807
MO000293886	EloC:EloB:Cul-2:Roc1:E2	352	808
MO000293887	HIF-1alpha:(RACK1{pS146})2:SSAT:EloC:EloB:Cul-2:Roc1:E2	352	808
MO000131926	ACLP-isoform1(h)	306	811
MO000131940	ACLP-isoform2(h)	306	811
MO000018695	SS1R(h)	397	818
MO000041133	(RANKL:RANK)3:(TRAF2:ASK1)2	500	826
MO000039194	(RANKL:RANK)3:traf6:tab2:TAB1:TAK1	500	845
MO000480195	glutamate decarboxylase 1(h){pT91}	339	846
MO000091427	glutamate decarboxylase 1(h)	339	847
MO000254900	glutamate decarboxylase 1-isoform3(h)	339	847
MO000327685	glutamate decarboxylase 1-isoform4(h)	339	847
MO000091426	glutamate decarboxylase 1-isoform1(h)	339	850
MO000145055	IFIT-1(h)	351	853
MO000061041	FcgammaRIIA(h)	462	869
MO000083712	fibronectin-xbb13(h)	405	869
MO000090105	fibronectin-1(h)	405	869
MO000090106	fibronectin-2(h)	405	869
MO000090107	fibronectin-3(h)	405	869
MO000090108	fibronectin-4(h)	405	869
MO000090109	fibronectin-5(h)	405	869
MO000090110	fibronectin-6(h)	405	869
MO000090111	fibronectin-7(h)	405	869
MO000090112	fibronectin-8(h)	405	869
MO000090113	fibronectin-9(h)	405	869
MO000090114	fibronectin-10(h)	405	869
MO000090115	fibronectin-11(h)	405	869
MO000090116	fibronectin-12(h)	405	869
MO000258977	fibronectin-isoform13(h)	405	869
MO000258978	fibronectin-isoform14(h)	405	869
MO000258979	fibronectin-isoform15(h)	405	869
MO000312129	fibronectin-isoform16(h)	405	869
MO000312130	fibronectin-isoform17(h)	405	869
MO000111873	NEC2-isoform1(h)	457	871
MO000096009	creatine kinase B-type(h)	485	891
MO000128217	IGFBP-7-isoform1(h)	464	893
MO000018602	edg3(h)	400	895
MO000059050	PAR2(h){ub}	441	895
MO000018683	PAR2(h)	441	900
MO000019400	RANK(h)	500	904
MO000022273	C5aR(h)	455	918
MO000036097	prolactin(h)	440	923
MO000204315	ASCL2(h)	498	925
MO000204316	ASH-2(h)	498	926
MO000061509	IGB(h)	458	951
MO000087906	SOD2(h)	494	954

Supplementary Table 5B. Master molecules found in RSS2 and their ranks.

RSS3 Master Molecules			
ID	Master molecule name	CMA rank	Total rank
MO000019826	NAP-2(h)	1	5
MO000214069	LGI1-isoform1(h)	2	8
MO000214071	LGI1-isoform2(h)	2	8
MO000214072	LGI1-isoform3(h)	2	8
MO000214070	LGI1(h)	2	11
MO000007287	inhibin beta A(h)	12	13
MO000141169	lithostathine1alpha(h)	10	15
MO000089119	IL-11-isoform1(h)	18	20
MO000128200	claudin2(h)	9	20
MO000020480	activin A(h)	12	26
MO000165657	activinA(h)	12	27
MO000019425	IL-11(h)	18	35
MO000312209	IL-11-isoform2(h)	18	36
MO000070193	OSR1(h)	21	37
MO000143095	ESM-1-isoform1(h)	6	38
MO000271046	ESM-1-isoform2(h)	6	38
MO000110915	Matrin(h)	37	40
MO000143096	ESM-1(h)	6	40
MO000189677	RENOX-isoform1(h)	24	43
MO000189679	RENOX-isoform2(h)	24	43
MO000189680	RENOX-isoform3(h)	24	43
MO000189681	RENOX-isoform4(h)	24	43
MO000189682	RENOX-isoform5(h)	24	43
MO000189684	RENOX-isoform7(h)	24	43
MO000328004	RENOX-isoform8(h)	24	43
MO000328005	RENOX-isoform9(h)	24	43
MO000145360	SEMP1(h)	34	44
MO000129931	MMP-13(h)	11	46
MO000145361	claudin1(h)	34	46
MO000039028	activin A:(ActR-II:ActR-IB{pS}{pT})2	12	48
MO000039029	activin A:(ActR-II:ActR-IB)2	12	49
MO000039030	activin A:(ActR-II)2	12	50
MO000110916	Matrin(h)	37	50
MO000038356	Fz:Wnt	22	51
MO000189683	RENOX-isoform6(h)	24	51
MO000189678	RENOX(h)	24	52
MO000166304	wnt2(h)	22	53
MO000129279	apolipoprotein-H(h)	36	66

Supplementary Table 5C. Master molecules found in RSS3 and their ranks.

GSVA Geneset Lists	
Immune Response	GO:0006955
Angiogenesis	GO:0001525
Stem Cell Proliferation	GO:0072089
Response to ER Stress	GO:0034976
Inflammatory Response	M5932
IL6-JAK-STAT3	M5897
G2M Checkpoint	M5901
DNA Repair	M5898
MYC2 targets	M5928
MYC1 targets	M5926
KRAS Signalling Up	M5953
WNT bCatening	M5895
Cell cycle, mitotic	M5336
EMT	M5930
Translation	M8229
ER UPR	M22993
Glycolysis	hsa00010
Pentose Phosphate Pathway	hsa00030
Fructose and Mannose Metabolism	hsa00051
Starch and Sucrose Metabolism	hsa00500
Galactose Metabolism	hsa00052
Nitrogen Metabolism	hsa00910
Tyrosine Metabolism	hsa00350
Glycerophospholipid Metabolism	hsa00564
Fatty Acid Degradation	hsa00071
Linoleic Acid Metabolism	hsa00591
Cancer Stem Cell EPHB2 signature	https://doi.org/10.1016/j.stem.2011.02.020
Cancer Stem Cell LGH5 signature	
Colonic Crypt late transit amplyfing	
Colonic Crypt, proliferative	

Supplementary Table 5D. Genesets used for GSVA and corresponding identifiers.
